# Interorgan communication in neurogenic heterotopic ossification: the role of brain-derived extracellular vesicles

**DOI:** 10.1038/s41413-023-00310-8

**Published:** 2024-02-22

**Authors:** Weicheng Lu, Jianfei Yan, Chenyu Wang, Wenpin Qin, Xiaoxiao Han, Zixuan Qin, Yu Wei, Haoqing Xu, Jialu Gao, Changhe Gao, Tao Ye, Franklin R. Tay, Lina Niu, Kai Jiao

**Affiliations:** 1 Department of Stomatology, Tangdu Hospital & State Key Laboratory of Oral and Maxillofacial Reconstruction and Regeneration & School of Stomatology, The Fourth Military Medical University, Xi’an, Shaanxi China; 2 State Key Laboratory of Oral and Maxillofacial Reconstruction and Regeneration & National Clinical Research Center for Oral Diseases & Shaanxi Key Laboratory of Stomatology, School of Stomatology, The Fourth Military Medical University, Xi’an, Shaanxi China; 3 The Dental College of Georgia, Augusta University, Augusta, GA USA

**Keywords:** Bone, Pathogenesis

## Abstract

Brain-derived extracellular vesicles participate in interorgan communication after traumatic brain injury by transporting pathogens to initiate secondary injury. Inflammasome-related proteins encapsulated in brain-derived extracellular vesicles can cross the blood‒brain barrier to reach distal tissues. These proteins initiate inflammatory dysfunction, such as neurogenic heterotopic ossification. This recurrent condition is highly debilitating to patients because of its relatively unknown pathogenesis and the lack of effective prophylactic intervention strategies. Accordingly, a rat model of neurogenic heterotopic ossification induced by combined traumatic brain injury and achillotenotomy was developed to address these two issues. Histological examination of the injured tendon revealed the coexistence of ectopic calcification and fibroblast pyroptosis. The relationships among brain-derived extracellular vesicles, fibroblast pyroptosis and ectopic calcification were further investigated in vitro and in vivo. Intravenous injection of the pyroptosis inhibitor Ac-YVAD-cmk reversed the development of neurogenic heterotopic ossification in vivo. The present work highlights the role of brain-derived extracellular vesicles in the pathogenesis of neurogenic heterotopic ossification and offers a potential strategy for preventing neurogenic heterotopic ossification after traumatic brain injury.

**Figure:**
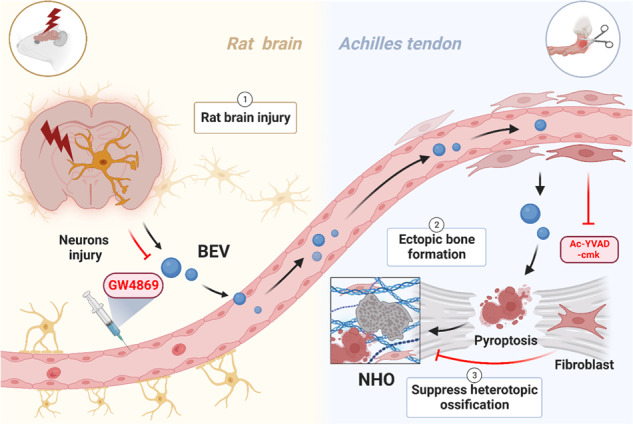
Brain-derived extracellular vesicles (BEVs) are released after traumatic brain injury. These BEVs contain pathogens and participate in interorgan communication to initiate secondary injury in distal tissues. After achillotenotomy, the phagocytosis of BEVs by fibroblasts induces pyroptosis, which is a highly inflammatory form of lytic programmed cell death, in the injured tendon. Fibroblast pyroptosis leads to an increase in calcium and phosphorus concentrations and creates a microenvironment that promotes osteogenesis. Intravenous injection of the pyroptosis inhibitor Ac-YVAD-cmk suppressed fibroblast pyroptosis and effectively prevented the onset of heterotopic ossification after neuronal injury. The use of a pyroptosis inhibitor represents a potential strategy for the treatment of neurogenic heterotopic ossification.

Brain-derived extracellular vesicles (BEVs) are released after traumatic brain injury. These BEVs contain pathogens and participate in interorgan communication to initiate secondary injury in distal tissues. After achillotenotomy, the phagocytosis of BEVs by fibroblasts induces pyroptosis, which is a highly inflammatory form of lytic programmed cell death, in the injured tendon. Fibroblast pyroptosis leads to an increase in calcium and phosphorus concentrations and creates a microenvironment that promotes osteogenesis. Intravenous injection of the pyroptosis inhibitor Ac-YVAD-cmk suppressed fibroblast pyroptosis and effectively prevented the onset of heterotopic ossification after neuronal injury. The use of a pyroptosis inhibitor represents a potential strategy for the treatment of neurogenic heterotopic ossification.

## Introduction

Different organs in multicellular organisms communicate with one another via a plethora of bioactive molecules.^1,2^ This form of communication helps to coordinate the physiological function of different organs under pathological conditions.^3,4^ Extracellular vesicles (EVs) are compartments formed by a lipid bilayer. The lipid bilayer separates the contents of EVs from the fluid-based extracellular environment and helps to preserve vesicular stability. EVs are important vectors for distant intercellular exchange and act as pathological mediators between different organs.^5,6^ Brain-derived extracellular vesicles (BEVs) are released after traumatic brain injury and can cross the blood‒brain barrier to participate in secondary injuries to distal organs such as the lung and kidney.^7,8^ The mechanism of BEV-induced distal injury after traumatic brain injury (TBI) has not been fully elucidated.^9^ Identifying pathological cues during BEV-induced distal injury is important for understanding the mechanism of interorgan communication.^10^

Neurological heterotopic ossification (NHO) is a common secondary complication that occurs after traumatic brain injury. This form of ossification is characterized by the abnormal formation of calcified nodules and even ectopic bone in injured periarticular tissues.^11^ Compared with general heterotopic ossification, NHO represents a more severe type of injury that causes substantial pain in patients and severely restricts their movement.^12^ Although current drug therapies, such as NSAIDs and bisphosphonates, radiation therapy and physical therapy, have been reported to be effective in managing NHO development, surgical intervention for explant ectopic bone is the only treatment option once the lesion has mineralized. Even after surgery, the rate of recurrence can reach 6%.^13–15^ There is an immediate need to understand the pathogenesis of NHO to design appropriate prophylactic interventions for this condition.^11^ The manifestation of NHO is closely related to the transmission of injury signals from the central nervous system to periarticular tissues.^11^ Brain-derived extracellular vesicles containing inflammatory proteins can be found in the blood after traumatic brain injury. After being phagocytosed by peripheral tissue cells, BEVs induce cell death and cause tissue damage.^16^ It has been reported that overexpression of *NOD*-like receptor family pyrin domain containing 3 (NLRP3) in injured tissues promotes heterotopic ossificaiton.^17^ Activation of NLRP3 induces pyroptosis, a highly inflammatory form of lytic programmed cell death. This, in turn, exacerbates inflammation and causes uncontrollable damage. There is no evidence in the current literature supporting the involvement of BEVs in NHO. In addition, whether BEVs carry inflammatory proteins that activate NLRP3 in distal injured tissues is unknown.

A rat model of NHO in which traumatic brain injury was combined with achillotenotomy was designed to address the aforementioned knowledge gaps.^18^ In addition, BEVs isolated from the plasma of rats that had undergone traumatic brain injury were cocultured with fibroblasts in vitro to examine whether BEVs could activate pyroptosis-related pathways.^19^ To develop a prophylactic strategy for NHO, we intravenously injected a pyroptosis inhibitor in vivo to examine whether this treatment could prevent NHO. The null hypotheses tested were as follows: (1) BEVs are not involved in NHO that develops after traumatic brain injury, and (2) the use of a pyroptosis inhibitor does not prevent the onset of NHO in rats.

## Results

### Traumatic brain injury accelerates NHO

**Fig. 1:**
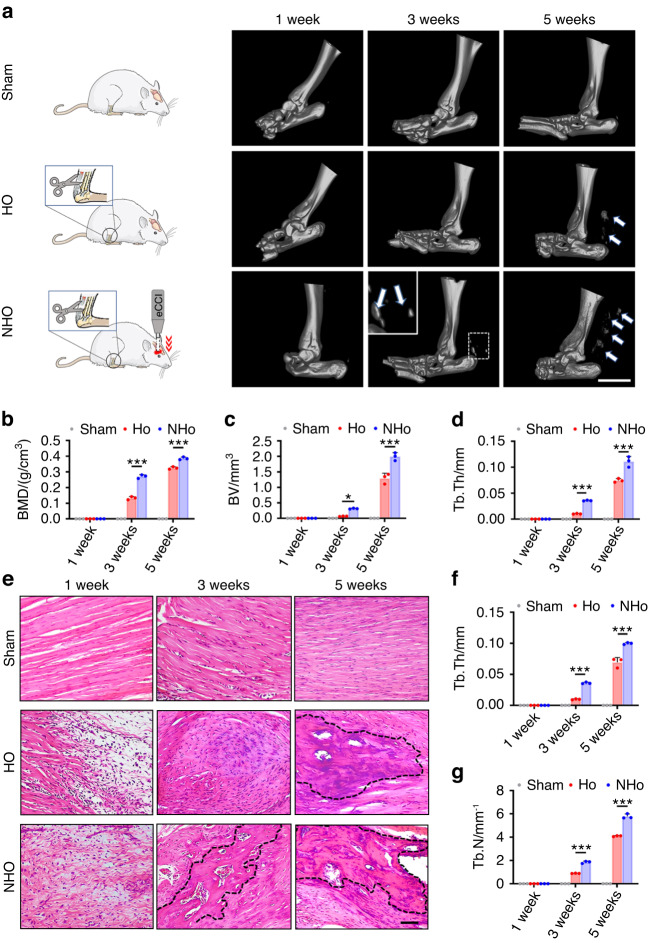
Traumatic brain injury intensifies the severity of NHO in a rat model. **a** Schematic and microcomputed tomography (micro-CT) images of the Achilles tendons of rats in the sham, HO and NHO groups at 1, 3 and 5 weeks. Heterotopic ossification was observed in the HO and NHO groups (white arrows in (**a**); eCCI electronic craniocerebral trauma instrument). Scale bar, 5 mm. **b**–**d** Quantitative analysis of bone histomorphometric parameters based on the micro-CT images in (**a**): **b** bone mineral density (BMD), **c** bone volume (BV) and **d** bone trabecular thickness (Tb. Th). **e** H&E staining of the Achilles tendons of rats in the sham, HO and NHO groups after 1, 3 and 5 weeks. Areas within the dotted black lines indicate bone trabecula. **f**, **g** Quantitative analysis of bone histomorphometric parameters based on H&E staining images in (**e**): **f** Tb.Th and **g** trabecular number (Tb.N). Scale bar, 100 μm. The data are presented as the means ± standard deviations (*n* = 3). Statistical analyses were performed by two-way ANOVA with post hoc Tukey’s test. *P < 0.05, ***P < 0.001

An electronic craniocerebral trauma instrument was used to control the severity of traumatic brain injury by monitoring the strength of cortical impact, and hematoxylin and eosin (H&E) staining of the damaged brain area showed significant cortical and hippocampal loss, as well as edema and distortion of the surrounding tissues (Fig. S1). Peripheral soft-tissue trauma was achieved by achillotenotomy.^18^ Microcomputed tomography (micro-CT) and H&E staining were used to examine the Achilles tendons of rats that underwent achillotenotomy only (HO group) or from those that underwent achillotenotomy after traumatic brain injury (NHO group). The rats in the sham group that were only subjected to a skin incision exhibited no heterotopic calcification in the Achilles tendon (Fig. 1a). There were a few cases of heterotopic calcification in the HO group. Heterotopic calcification was observed in the NHO group 3 weeks after surgery (Fig. 1a). After 5 weeks, more ectopic bone nodules could be identified in Achilles tendons derived from the HO group and the NHO group, and after 12 weeks, fully developed cancellous bone had formed in the trauma area in the HO and NHO groups, while the control group had no HO lesions (Fig. 1a and Fig. S2). There was significantly more ectopic bone in the NHO group than in the HO group at the same time points (*P* < 0.05; Fig. 1a). Quantitative analysis of bone mineral density, bone volume and trabecular thickness revealed an increase in bone mass in the NHO group (Fig. 1b–d). Similar to those in the sham group, rats in the HO and NHO groups exhibited increases in weight, and there were no significant differences among the sham, HO and NHO groups (Fig. S3). These data suggest that, unlike general heterotopic ossification, TBI accelerates heterotopic ossification in the NHO group.

H&E staining showed inflammatory cell infiltration in the injury site without the formation of osteoids in the HO and NHO groups after 1 week (Fig. 1e). After 3 weeks, nonossified osteoids were found in the HO group, whereas heterotopic ossification was observed in the NHO group (Fig. 1e). Safranin O and fast green (SOFG) staining revealed cartilage layers in the HO group and thick proteoglycan-enriched cartilage layers adjacent to cancellous bone in the NHO group, and the positive area in the NHO group was significantly larger than that in the HO group (*P* < 0.05, Fig. S4). After 5 weeks, ectopic bone nodules with marrow cavities were observed in the experimental groups, and analysis of the bone trabecular thickness and trabecular number confirmed that ectopic bone formation occurred earlier in the NHO group and that the extent of bone formation was greater (Fig. 1e–g).

In the HO and NHO groups, the immunofluorescence staining results showed that the major inflammatory cells in the injured tendon were macrophages, and the number of macrophages was significantly higher in the NHO group than in the HO group, while staining for T cells and neutrophils was negative. The stained Achilles tendons in the sham group did not show inflammatory cell infiltration (Fig. S5). Immunostaining for Nestin revealed a significantly more Nestin^+^ cells in the NHO group than in the HO group, and the staining of Nestin was almost negative in the sham group (Fig. S6). The negative controls also demonstrated that the positive staining in Figs. S5 and S6 was not an artifact. We also performed Alizarin red staining to measure the calcification of tissues at 1 week in the sham, HO and NHO groups. Alizarin red S staining indicated the presence of minerals at 1 week in the HO group, and these levels were increased in the NHO group. We also evaluated tendon mineral distribution and morphology by TEM, and the results showed that calcification was widely dispersed in tendon ECM in the NHO group at 1 week (Fig. S7). Taken together, these results indicated that heterotopic ossification within the injured tendon was accelerated and intensified after traumatic brain injury.

### Characterization and isolation of BEVs in the NHO group

**Fig. 2:**
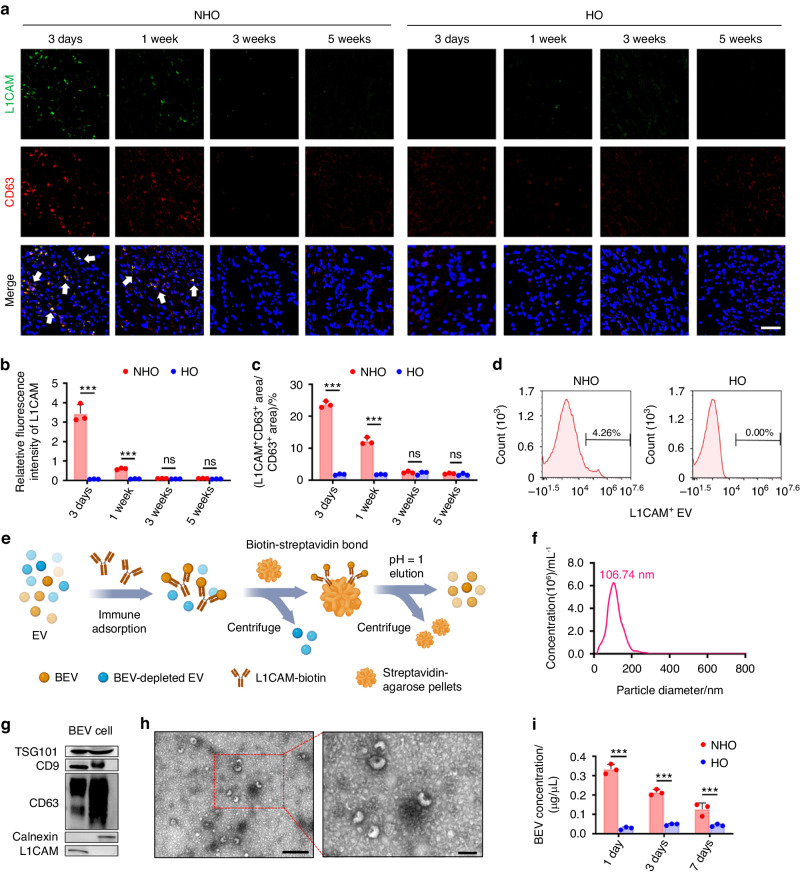
Characterization and isolation of BEVs in NHO. **a** Immunofluorescence images showing the localization of L1CAM (green) and CD63 (red) in the Achilles tendons of rats in the HO and NHO groups after 3 days and after 1, 3 and 5 weeks. BEVs are indicated by arrows. Scale bar: 100 μm. **b** Quantitative analysis of the relative fluorescence intensity of L1CAM in each group. **c** Quantitative analysis of the percentage of the L1CAM^+^ CD63^+^ area in the CD63^+^ areas from different groups. **d** Flow cytometric analysis of L1CAM^+^ BEVs in plasma after surgery. **e** Schematic representation of BEVs isolated from the plasma of rats in the NHO group. **f** Nanoparticle tracking analyses of BEVs from rat plasma derived from the NHO group after 3 days. **g** Western blot showing the expression of TSG101, CD9, CD63, calnexin and L1CAM in BEVs from the plasma and fibroblasts of rats in the NHO group after 3 days. **h** TEM images of BEVs from the plasma of rats in the NHO group after 3 days. Scale bar: 500 nm. High magnification of the area in the red rectangle in the low magnification image. Scale bar: 100 nm. **i** Quantification of plasma BEV concentrations in rats in the HO and NHO groups after 1, 3 and 7 days. The data are presented as the means ± standard deviations (*n* = 3). Statistical analyses were performed by two-way ANOVA with post hoc Tukey’s test. ns, no significance. ***P < 0.001

Previous studies have reported that inflammation following traumatic brain injury persists for a long time after the primary insult.^20^ In this regard, BEVs play a pivotal role in worsening inflammation in distal tissues.^21^ To determine whether BEVs were involved in exacerbating postinjury inflammation, we labeled CD63 (a marker of EVs) with L1 cell adhesion molecule (L1CAM, a marker of the neural region) and characterized BEVs in tendons derived from the different groups. L1CAM and CD63 double-positive BEVs were identified in the injured tendons of the NHO group (Fig. 2a). The quantity of BEVs in the tendons of the NHO group peaked 3 days after injury and declined gradually with time. The quantity of BEVs was significantly higher in the 3-day and 1-week NHO groups than in the time-matched HO groups (*P* < 0.05). Few BEVs were observed in the HO and NHO groups (at 3 and 5 weeks), and no significant differences were observed between the NHO and HO groups at 3 and 5 weeks postinjury (Fig. 2b, c). To clarify the reason why BEVs were drawn to sites of injury, we conducted experiments on NHO rats in which the Achilles tendon of the left leg was sham-treated (sham) and that of the right leg was injured (injured). The results showed that the injured right tendon was more vulnerable to ossification than the left tendon (sham) after brain injury, and more importantly, the number of BEVs was significantly higher in the injured tendon than in the sham-treated tendon (Fig. S8). These in vivo results demonstrated that the BEVs increasingly accumulated at sites of injury, which was closely related to the increase in local ossification at the early stage and subsequent heterotopic bone formation. The flow cytometry results also indicated that the percentage of L1CAM^+^ EVs in the plasma of the NHO group was higher than that in the plasma of the HO group 1 day after TBI (Fig. 2d). These findings also indicated that the concentration of BEVs in the injured tendon peaked at the onset of inflammation and gradually decreased as the inflammatory response subsided.

To examine the changes in BEV concentrations after TBI in vivo, EVs were first isolated from the plasma of the rats via ultracentrifugation. The BEVs present among the isolated EVs were labeled with biotinylated L1CAM and harvested via a biotin-streptavidin reaction using streptavidin-coated agarose pellets (Fig. 2e). The morphology of the harvested BEVs and their specific proteins were characterized according to the proposal of the International Society of Extracellular Vesicles.^22^ Nanoparticle tracking analysis revealed that the diameter of the BEVs was 106.7 ± 10.4 nm, which was consistent with that of exosomes (40–150 nm) (Fig. 2f).^23^ Western blot analysis confirmed the presence of the exosome markers TSG101, CD9 and CD63 and the negative control marker calnexin. The neural marker L1CAM was present in BEVs (Fig. 2g). Transmission electron microscopy (TEM) revealed that the BEVs were tea cup-shaped and approximately 110 nm in diameter (Fig. 2h).

Plasma BEV concentrations 1, 3 and 7 days after surgery were detected by flow cytometry and a bicinchoninic acid (BCA) protein assay.^24^ The changes in plasma BEV concentrations were consistent with the changes in BEV concentrations in the injured tendon (Fig. 2a). As determined by the BCA method, plasma BEV concentrations in the NHO group were considerably higher than those in the HO group (Fig. 2i). The results showed that the concentration of BEVs in the brain was significantly greater in the NHO group than in the HO group at 1, 3 and 7 days postinjury (*P* < 0.05). Thus, the differences in BEV concentrations between the HO and NHO groups were consistent in the brain and plasma (Fig. S9). Taken together, the in vivo results indicated that BEVs, which are released into the blood after traumatic brain injury, can accumulate in distal injured tissues. Because the increase in BEV concentrations within distal tissues coincided with the development of a local inflammatory response in NHO patients, there may be a link between BEVs and heterotopic calcification.

### BEVs are inducers of NHO

**Fig. 3:**
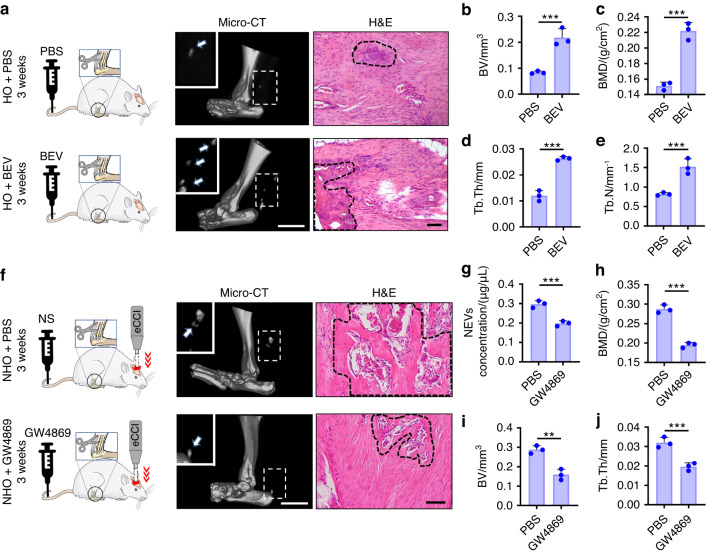
BEVs as inducers of NHO. **a** Schematic representation, micro-CT images (scale bars: 5 mm) and H&E staining (scale bars: 100 μm) of heterotopic ossification in rats that underwent achillotenotomy in the PBS and BEV injection groups. eCCI: electronic craniocerebral trauma instrument**. b**–**e** Quantification of BV (**b**), BMD (**c**), Tb. Th (**d**) and Tb. N (**e**) based on data derived from the micro-CT images in (**a**). **f** Schematic representation, micro-CT images (scale bars: 5 mm) and H&E staining (scale bars: 100 μm) of HO formation in the rats with achillotenotomy in the normal saline (NS) and GW4869 injection groups. **g** Quantification of the concentration of BEVs isolated from the plasma of rats injected with NS or GW4869. **h**–**j** Quantification of BMD (**h**), BV (**i**) and Tb.Th (**j**) based on the data derived from the micro-CT images in (**f**). The data are presented as the means ± standard deviations (*n* = 3). Statistical analyses were performed using Student’s *t*-test. **P < 0.01, ***P < 0.001

To determine the relationship between BEVs and NHO, BEVs were first isolated from the plasma of rats that had traumatic brain injury. These BEVs were intravenously injected into rats that had undergone achillotenotomy to examine whether heterotopic calcification was aggravated after BEV injection. Three weeks after the injection, the micro-CT results indicated that heterotopic calcification was more common in rats that were injected with BEV than in those that were injected with PBS (Fig. 3a). These findings were validated by histomorphometric analysis: the BV and BMD values in rats injected with BEV were significantly greater than those in rats injected with PBS (Fig. 3b, c). The increases in Tb.Th and Tb.N in rats injected with BEV were indicative of an increase in trabecular thickness (Fig. 3d, e). H&E staining of the Achilles tendon showed that more profuse heterotopic calcification occurred in rats injected with BEV (Fig. 3a). As a negative control, GW4869, which is an inhibitor of EV formation, was injected into rats with traumatic brain injury and achillotenotomy through the tail vein.^25^ The BCA assay results revealed a significant reduction in the number of BEVs isolated from rats injected with GW4869 (Fig. 3g). There were significant reductions in BMD and BV values after GW4869 injection (Fig. 3f, h, i). The trabecular indicator Tb.Th was also significantly reduced after GW4869 injection (Fig. 3j). Taken together, these results indicate that BEVs are inducers of NHO.

### Identification of the contents of BEVs

**Fig. 4:**
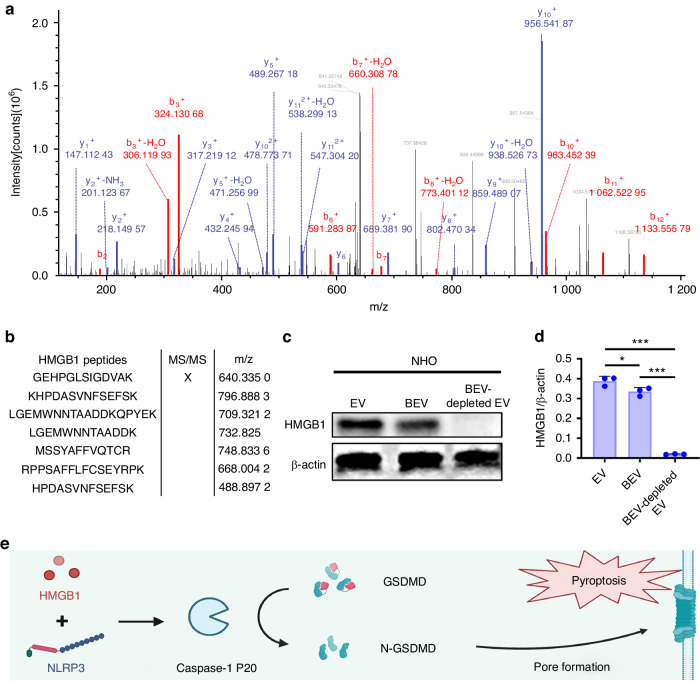
Identification of the contents of BEVs. **a** Electrospray ionization mass spectrum of BEV proteins showing the ions observed in the m/z range of 100–1 200. **b** Observed masses in the mass spectrum corresponded in mass to the tryptic peptide of the HMGB1 protein. The “X” symbol indicates the m/z 640.335 01 region analyzed in (**a**). **c** Protein expression of HMGB1 in EVs, BEVs and BEV-depleted EVs. **d** Quantitative analysis of the protein expression of HMGB1/β-actin. **e** Schematic representation of pyroptosis induced by HMGB1. The data are presented as the means ± standard deviations (*n* = 3). Statistical analyses were performed using one-way ANOVA with post hoc Tukey’s test. *P < 0.05, ***P < 0.001

Mass spectrometry (MS) was used to identify the intravesicular proteins involved in this disease.^26^ The MS/MS spectrum showed a series of ions that were derived from the fragmentation of the peptide backbone. These ions were in the mass range of 100–1 800. The MS/MS spectrum was evaluated with an MS data analysis program (SequestHT) and identified the sequence of the peptide as GEHPGLSIGDVAK. The sequence corresponded to a tryptic peptide (residues 114–128) of rat high mobility group protein 1 (HMGB1) (Fig. 4a).^27^ There were seven of these ions that corresponded with HMGB1 peptides (Fig. 4b and Fig. S10). In contrast, we did not find HMGB1 in the plasma EVs of rats in the HO groups (Table S1). We measured the level of HMGB1 in total EVs, BEVs and BEV-depleted EVs, and HMGB1 protein was derived predominantly from BEVs (Fig. 4c, d).

The HMGB1 protein is a damage-associated molecular pattern (DAMP) molecule. It is cytotoxic and causes pyroptosis and tissue injury.^28,29^ Pyroptosis is triggered by the NLRP3 inflammasome, which can sense DAMPs such as HMGB1. Activation of NLRP3 causes the activation of cysteinyl aspartate specific proteinase-1 (CASPASE-1), which cleaves gasdermin-D (GSDMD) into two fragments. The N-GSDMD fragment oligomerizes in the plasma membrane to create pores that increase membrane permeability, resulting in pyroptosis and the release of interleukin-1 beta (Fig. 4e).^30^ Histological analysis of the pyroptosis-related proteins CASPASE-1 and N-GSDMD was performed in subsequent experiments.

### BEVs promote fibroblast pyroptosis in vivo

**Fig. 5:**
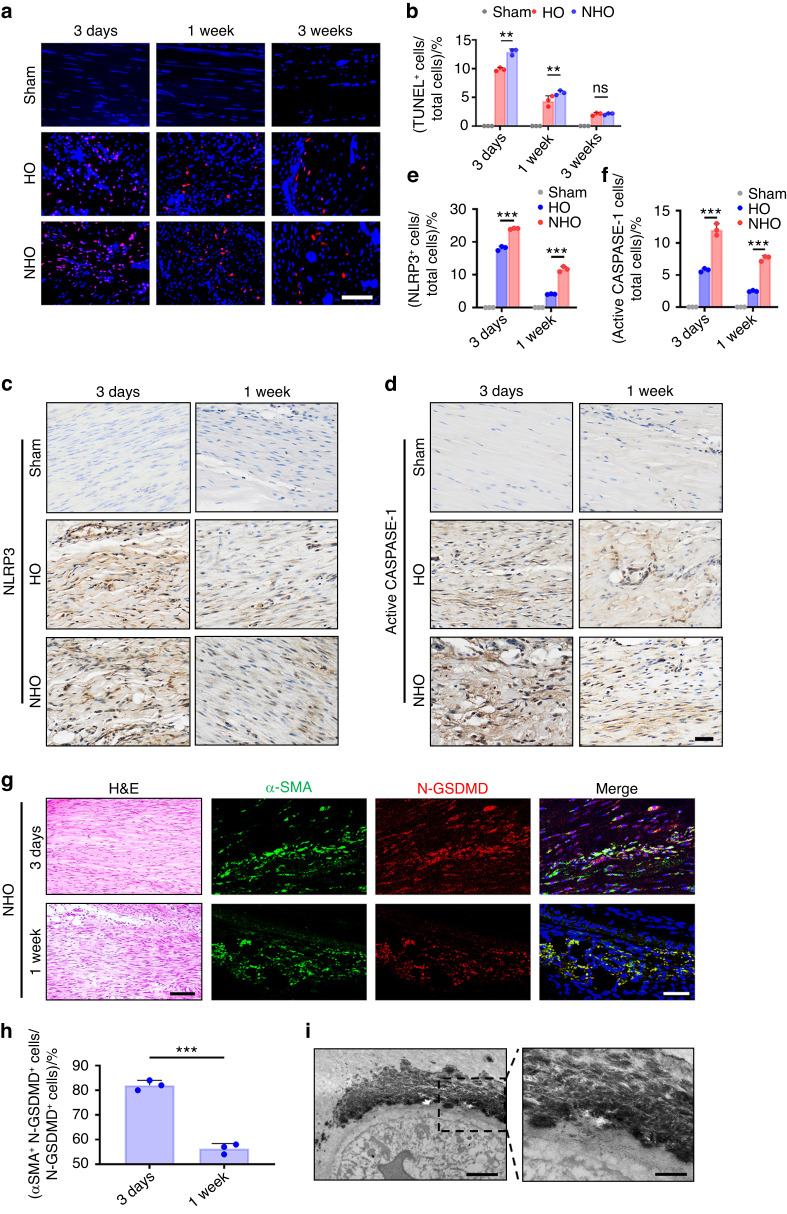
Traumatic brain injury promoted fibroblast pyroptosis in the Achilles tendon after achillotenotomy. **a** TUNEL staining of the Achilles tendons of rats in the sham, HO and NHO groups after 3 days and after 1 and 3 weeks. Scale bar, 100 μm. **b** Quantitative analysis of TUNE-positive cells in (**a**). **c**, **d** Representative images of immunohistochemical staining of Achilles tendons from the sham, HO and NHO groups after 3 days and 1 week. Scale bar, 50 μm. **e**, **f** Quantitative analysis of NLRP3^+^ cells and CASPASE-1 P20^+^ cells in (**c**) and (**d**). **g** Representative confocal images of N-GSDMD (red), α-SMA (green) and DAPI (blue) in rat tendons after traumatic brain injury and achillotenotomy. Scale bar, 50 μm. **h** Quantitative analysis of the percentage of α-SMA^+^ N-GSDMD^+^ cells (pyroptotic fibroblasts) among N-GSDMD^+^ cells (pyroptotic cells). **i** Representative TEM images of pyroptosis and calcium deposition. Scale bar: 500 nm. High magnification of the area depicted by the black rectangle in the low magnification image. Scale bar: 100 nm. The data are presented as the means ± standard deviations (*n* = 3). For (**b**), (**e**), and (**f**), statistical analyses were performed by two-way ANOVA with post hoc Tukey’s test. For (**h**), statistical analyses were performed by Student’s *t*-test. ns, no significance. *P < 0.05, **P < 0.01, ***P < 0.001

TUNEL staining of the injured Achilles tendon after traumatic brain injury in vivo revealed dead cells in the injured tissues. The number of dead cells decreased over time in the NHO group (Fig. 5a, b). There were more NLRP3- and CASPASE-1 P20-positive cells in the injured Achilles tendon in the NHO group than in the HO group, and there were significant reductions in the number of positive cells over time in the HO and NHO groups (*P* < 0.05; Fig. 5c–f). These data confirmed that pyroptosis occurred when the Achilles tendon was injured after traumatic brain injury.

Morphological examination of the injured Achilles tendon revealed an early inflammatory response characterized by loose connective tissue formation that was gradually replaced by fibroblasts and a collagenous matrix.^31^ The authors hypothesized that the predominant cell type that resided in the injured areas and underwent pyroptosis were fibroblasts. To verify this hypothesis, the Achilles tendons of rats in the sham, HO and NHO groups were examined by immunofluorescence staining. There was colocalization of the fibroblast marker α-smooth muscle actin (α-SMA) and the pyroptosis marker N-GSDMD in the NHO group (Fig. 5g). In addition, there was a reduction in the percentage of pyroptotic fibroblasts (α-SMA^+^ N-GSDMD^+^) among all pyroptotic cells (N-GSDMD^+^) over time. There were more fibroblasts in the NHO group than in the HO group, and significantly more pyroptotic fibroblasts were observed in the NHO group than in the HO group (*P* < 0.05; Fig. 5g, h and Fig. S11). These findings suggested that fibroblast pyroptosis occurred only during the early stage of injury, despite the presence of fibroblasts in the Achilles tendon after injury. Calcium deposition was detected by TEM in the injured tendon 1 week after traumatic brain injury. Deposition occurred around membrane pores containing pyroptotic fibroblasts. This finding suggested a potential link between pyroptosis and heterotopic calcification (Fig. 5i).

### BEV uptake induces fibroblast pyroptosis in vitro

**Fig. 6:**
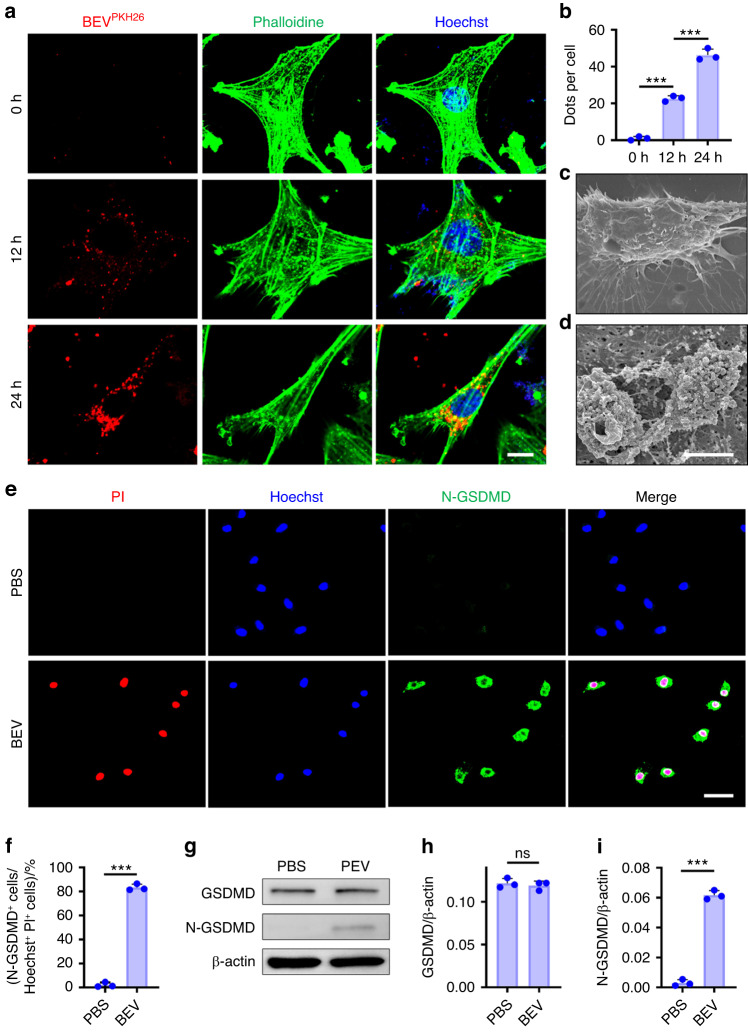
Engulfment of BEVs increased pyroptosis in fibroblasts. **a** Immunofluorescence staining of fibroblasts with PKH26 (red), phalloidin (green) and Hoechst (blue) after the cells were incubated with BEVs for 0, 12 and 24 h. Scale bar, 2 μm. **b** Quantitative analysis of BEVs (red dots) in fibroblasts. **c**, **d** SEM image of fibroblasts cultured with or without BEVs for 24 h. Scale bar, 2 μm. **e** Immunofluorescence images of fibroblasts stained with N-GSDMD (green), propidium iodide (PI; red) and Hoechst (blue) after the cells were incubated with or without BEVs for 24 h. Scale bar, 50 μm. **f** Quantitative analysis of the percentage of N-GSDMD^+^ cells among Hoechst^+^ PI^+^ cells. **g** Western blot showing the expression of N-GSDMD and GSDMD in fibroblasts. **h**, **i** Quantitative analysis of GSDMD and N-GSDMD expression in fibroblasts after they were cocultured with BEVs. The data are presented as the means ± standard deviations (*n* = 3). Statistical analyses were performed by Student’s *t*-test and one-way ANOVA with post hoc Tukey’s test. ns, no significance. ***P < 0.001

Further in vitro experiments were conducted to investigate how fibroblasts internalize BEVs and become pyroptotic. Immunofluorescence staining after the addition of PKH26-labeled BEVs to phalloidin-labeled fibroblasts revealed that the BEVs were engulfed by fibroblasts (Fig. 6a). Over time, the fibroblasts took up more BEVs (Fig. 6b). Fibroblasts that were cultured with BEVs underwent pyroptosis (Fig. 6e, f). Scanning electron microscopy (SEM) showed that the surface of normal cells was intact and devoid of membrane damage. In contrast, the fibroblasts that had taken up BEVs lost their membrane integrity and exhibited disorganization of the cytoplasm (Fig. 6c, d). Western blot analysis confirmed that the protein expression of the N-GSDMD increased after fibroblasts were cultured with BEVs for 24 h (Fig. 6g–i). These data further demonstrated that fibroblasts underwent pyroptosis after being cultured with BEVs.

### BEV uptake induces heterotopic calcification in vitro and in vivo

Previous studies have reported that persistent pyroptotic signaling induces ectopic ossification.^32^ To understand the effects of fibroblast pyroptosis on ectopic ossification, fibroblasts were cocultured with EVs derived from different origins in vitro. The experiment consisted of five groups: a control group (with PBS), an EV group (with total EVs isolated from plasma), a BEV group (with BEVs isolated from plasma), a BEV-depleted EV group (with surplus EVs after removal of BEVs from total plasma EVs), and a BEV plus CASPASE-1 inhibitor group (with BEVs and Ac-YVAD-cmk, a selective CASPASE-1 inhibitor).

**Fig. 7:**
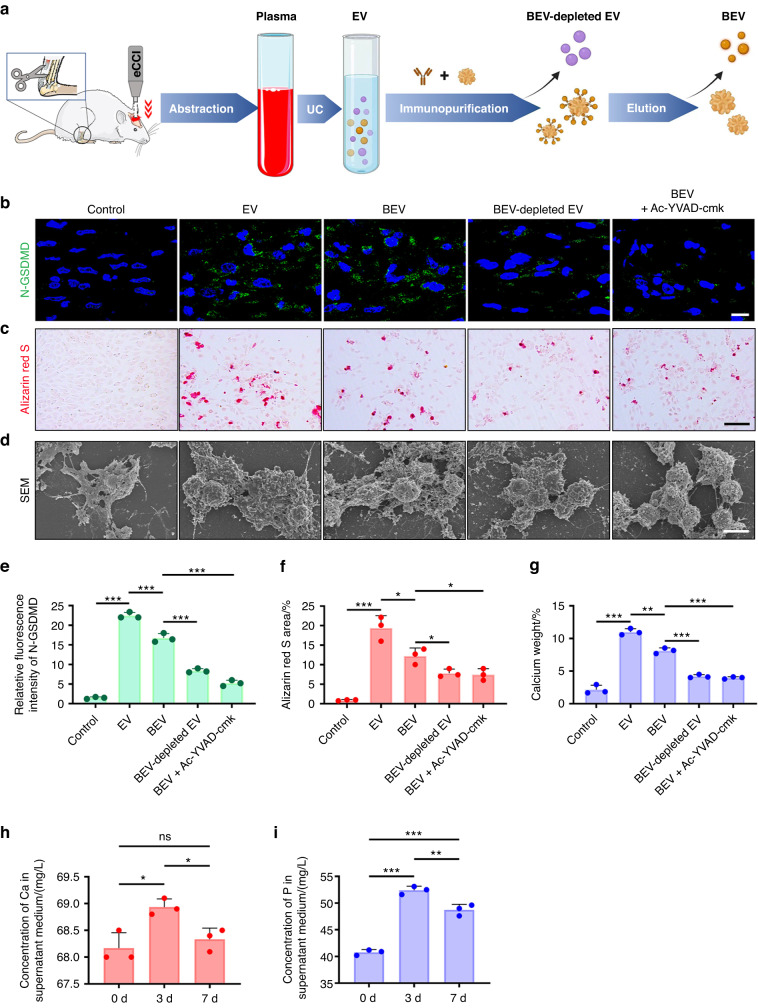
BEV uptake caused ectopic calcification in vitro. **a** Schematic representation of the extraction of different kinds of EVs isolated from plasma. **b** Immunofluorescence staining of N-GSDMD in fibroblasts incubated with plasma EVs, BEVs, plasma EV-depleted BEVs or BEVs plus a CASPASE-1 inhibitor (Ac-YVAD-cmk) for 24 h. Scale bar, 10 μm. **c** Alizarin red S staining of the calcium deposits in each group. Scale bar, 100 μm. **d** SEM of fibroblasts from different groups. Scale bar, 10 μm. **e**–**g** Quantitative analysis of the relative fluorescence intensity of N-GSDMD in (**b**), the alizarin red area in (**c**) and the weight of the calcium deposits in (**d**). **h**, **i** Concentration of calcium and phosphorus in the supernatant of fibroblasts incubated with BEVs for 0, 3 and 7 days. The data are presented as the means ± standard deviations (*n* = 3). Statistical analyses were performed using one-way ANOVA with post hoc Tukey’s test. ns, no significance. *P < 0.05, **P < 0.01, ***P < 0.001

Immunofluorescence staining of N-GSDMD was used to detect pyroptosis after fibroblasts were cultured as indicated in the different groups for 24 h. Alizarin red S was used to label calcium deposits after 7 days. The BEV group exhibited fibroblast pyroptosis and calcium deposition. However, compared to that in the EV group, the expression of N-GSDMD in the BEV group was lower, and the calcification area was smaller (Fig. 7a–c). BEV-depleted EVs play a role in inducing fibroblast pyroptosis and calcium deposition but are not as important as BEVs (Fig. 7e, f). The BEV plus CASPASE-1 inhibitor group exhibited markedly lower expression of N-GSDMD and less calcium deposition than the other groups (Fig. 7b, c, e, f), further validating the role of pyroptosis induced by BEVs in calcium deposition. Scanning electron microscopy confirmed that the plasma membranes of fibroblasts in the control group were intact. Calcium deposits mixed with dead cell fragments were observed in the EV group and the BEV group (Fig. 7d). The weights of the calcium deposits in the BEV-depleted EV group and the BEV plus CASPASE-1 inhibitor group were significantly lower than those in the EV and BEV groups (Fig. 7d, g). As calcium was deposited, the concentrations of calcium and phosphorus in the solution began to decrease (Fig. 7h, i). Taken together, these data suggest that fibroblast pyroptosis induced by BEVs leads to increases in calcium and phosphorus concentrations in fibroblasts. This, in turn, creates a microenvironment that promotes the deposition of calcium salts (Fig. S12).

**Fig. 8:**
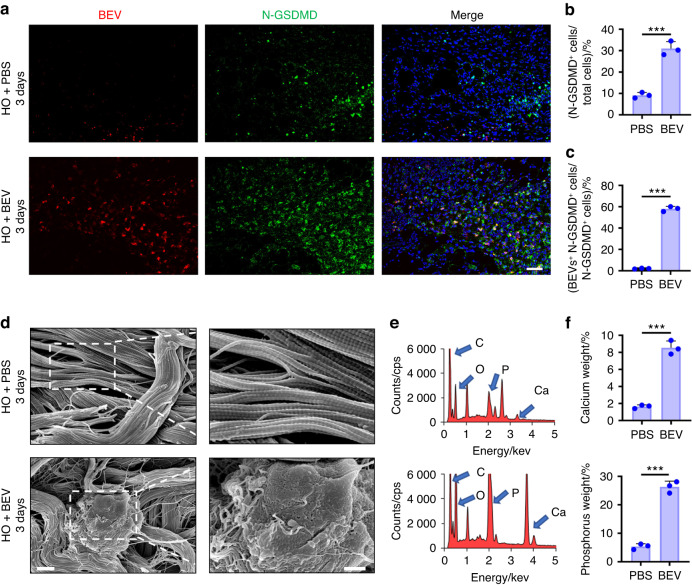
BEVs exacerbated pyroptosis and HO formation in rats subjected to achillotenotomy. **a** Representative confocal image of BEV (red), N-GSDMD (green) and DAPI (blue) in the Achilles tendons of rats 3 days after the injection of PKH26-labeled BEVs isolated from rats that had undergone traumatic brain injury or from rats that were injected with PBS only. Scale bars, 100 μm. **b**, **c** Quantitative analysis of the percentage of N-GSDMD^+^ cells among total cells and BEV^+^ N-GSDMD^+^ cells among N-GSDMD^+^ cells. **d** SEM image of the Achilles tendon 3 days after the injection of BEVs or PBS in rats. Scale bar, 1 μm (left), 300 nm (right). **e**, **f** Elemental analysis of the relative concentrations of Ca and P in the Achilles tendon in (**d**). The data are presented as the means ± standard deviations (*n* = 3). Statistical analyses were performed by Student’s *t*-test. ***P < 0.001

To validate the in vitro data, PKH26-labeled BEVs were injected into rats to verify fibroblast pyroptosis in the injured areas in the NHO group. The objective of the in vivo experiment was to determine whether local calcification was aggravated. The injured Achilles tendons were observed by immunofluorescence staining. After 3 days, BEVs were found in N-GSDMD-positive cells (Fig. 8a). The number of N-GSDMD-positive cells in the Achilles tendon was significantly increased after BEV injection (*P* < 0.05; Fig. 8b). Quantitative analysis of N-GSDMD-positive cells plus BEVs revealed that a high percentage (>70%) of pyroptotic fibroblasts were induced by BEV uptake (Fig. 8c).

Tendons were injected with BEVs or phosphate-buffered saline (PBS) to examine the mechanism through which BEVs induce NHO. Calcium salt deposition was observed in SEM images of tendons that were injected with BEVs (Fig. 8d). Elemental analysis revealed that the tendons contained calcium and phosphorus (Fig. 8e). The relative concentrations of calcium and phosphorus in the tendons of rats injected with BEVs were higher than those in the tendons of rats injected with PBS (Fig. 8f).

### Effect of injecting a pyroptosis inhibitor in vivo

**Fig. 9:**
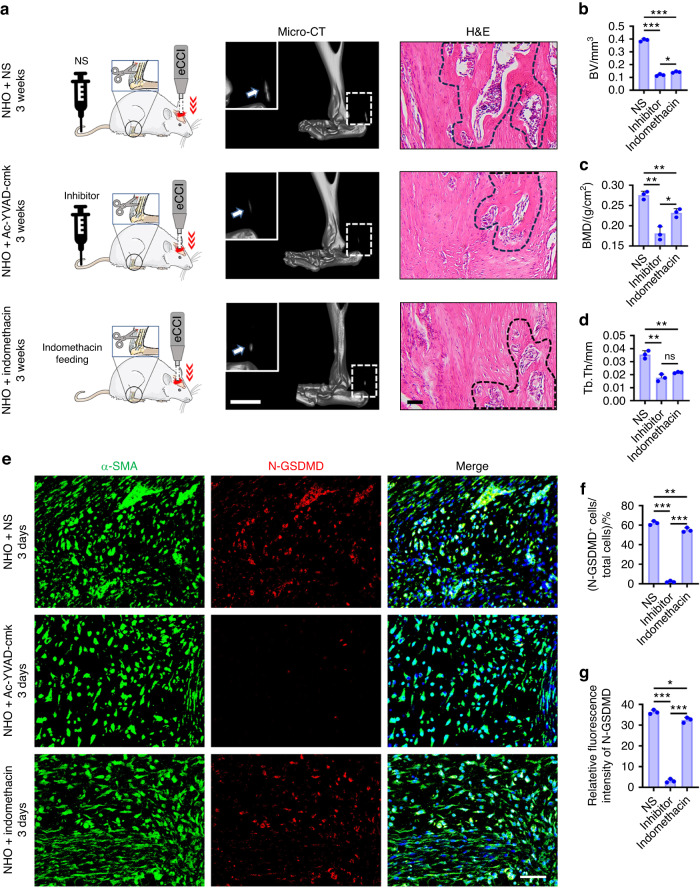
Ac-YVAD-cmk inhibited pyroptosis and prevented NHO development in rats. **a** Schematic and micro-CT images (scale bars, 5 mm) and H&E staining images (scale bars, 100 μm) of heterotopic calcification in the Achilles tendon 3 weeks after surgery. **b**–**d** Quantification of BV (**b**), BMD (**c**) and Tb.Th (**d**) HO formation based on the micro-CT images in (**a**). **e** Representative confocal images of Achilles tendons stained with α-SMA (green), N-GSDMD (red) and DAPI (blue) in the rat tendons 3 days after surgery. Scale bars, 100 μm. **f**, **g** Quantitative analysis of the percentage of N-GSDMD^+^ cells relative to total cells and the relative fluorescence intensity of N-GSDMD. The data are presented as the means ± standard deviations (*n* = 3). Statistical analyses were performed using one-way ANOVA with post hoc Tukey’s test. ns, no significance. *P < 0.05, **P < 0.01, ***P < 0.001

The selective caspase-1 inhibitor Ac-YVAD-cmk was used to examine its potential role in inhibiting NHO. Ac-YVAD-cmk was injected intravenously into rats subjected to traumatic brain injury and achillotenotomy. Micro-CT of the rats at 3 weeks after the injection revealed significant decreases in the bone volume (BV), bone mineral density (BMD), and trabecular thickness (Tb.Th) in the Ac-YVAD-cmk group (Fig. 9a–d). Immunofluorescence images showed that fibroblast pyroptosis in the Achilles tendon was inhibited 3 days after postsurgical injection of Ac-YVAD-cmk (Fig. 9e). Quantitative analysis of N-GSDMD-positive cells revealed that >60% of the fibroblasts had undergone pyroptosis (Fig. 9f). Ac-YVAD-cmk inhibited fibroblast pyroptosis (Fig. 9f). Indomethacin, an NSAID analgesic, was used as a positive control for NHO treatment. After 3 weeks of indomethacin treatment, micro-CT revealed significant decreases in the BV, BMD and Tb.Th compared to those in the NS group. The number of BEVs in the indomethacin group was significantly lower than that in the NS and Ac-YVAD-cmk groups. These in vivo results demonstrated that indomethacin inhibited fibroblast pyroptosis by suppressing the accumulation of BEVs at sites of injury (Fig. 9e–g and Fig. S13). Taken together, these results suggested that inhibiting fibroblast pyroptosis in injured tendons could prevent NHO development in this small animal model.

## Discussion

Studies of interorgan communications have shown the existence and importance of EVs that mediate communication between organs by transmitting factors such as signaling proteins.^33^ These factors coordinate the function of distal organs in physiological (e.g., starvation, high-fat diet) and disease states.^34^ Neurological heterotopic ossification is a form of secondary injury that occurs after traumatic brain injury and is associated with greater severity and a higher incidence of reoccurrence than general HO.^11^ Nerve-derived biological factors have been reported to play important roles in NHO. However, the mechanism underlying this phenomenon is unknown.^35^ The present work demonstrated that traumatic brain injury expedited NHO through the release of BEVs and aggravated the severity of the injured tendon. These results suggested that the injured brain communicated with extra cranial soft-tissue injury through BEVs.

The DAMP molecule HMGB1 was present in BEVs isolated from the plasma of rats after traumatic brain injury. HMGB1 senses NLRP3 and activates pyroptosis-related proteins such as CASPASE-1 and N-GSDMD.^28^ Clinical evidence has shown that TBI can accelerate bone fracture healing and increase the amount of callus,^25^ and early after TBI, plasma exosomes can promote osteoblast proliferation and differentiation.^36^ The present results demonstrate that HMGB1 plays a role in inflammasome activation and induces fibroblast pyroptosis during NHO, and it has also been reported that HMGB1 can accelerate the healing of fractures by promoting the expression of osteogenesis-related genes.^37–39^ However, whether HMGB1-related signaling pathways are involved in TBI-induced bone fracture is still unclear and needs further study. BEVs also carry cytokines, autoantigens and tissue-degrading enzymes, such as proteases and glycosidases.^40^ A recent study showed that proinflammatory cytokines, including TNF-α, IL-1β, IL-6, S-100B, E-selectin, and caspase-1, are released after TBI, taken up by lung cells and trigger local inflammation.^40^ Although our results confirmed that HMGB1 plays a role in inflammasome activation in NHO, other molecules within BEVs may be involved in inflammasome activation and need to be further explored.

Previous research has shown that downregulation of the NLRP3 inflammasome prevents heterotopic calcification in HO, suggesting a critical role for pyroptosis in the pathogenesis of HO.^41,42^ However, whether pyroptosis is related to NHO has yet to be determined. By analyzing the location of CASPASE-1 and N-GSDMD, fibroblasts were shown to be the predominant cells that underwent pyroptosis in the injured tendon. In vitro experiments confirmed that the engulfment of BEVs induced fibroblast pyroptosis. In vivo injection of BEVs promoted fibroblast pyroptosis and aggravated heterotopic calcification in injured tendons even in the absence of traumatic brain injury. Although BEVs induce fibroblast pyroptosis in vitro and in vivo, how pyroptosis induces calcium deposition has not been determined. The development of HO in patients with TBI has a multifactorial etiology involving the interaction of several local (traumatic), neurogenic and systemic factors.^43^ Although central nervous system damage has been identified as an important risk factor for HO, increasing evidence has demonstrated that HO is positively correlated with the severity of local trauma to joints and muscles.^44^ We found that the BEVs increasingly accumulated at sites of injury, which was closely related to the increase in local ossification at the early stage and subsequent heterotopic bone formation. The ability of EVs to accumulate at injured sites has also been verified by in vivo imaging of EVs in animal models of liver regeneration and renal ischemia‒reperfusion injury.^45,46^ EVs exert extensive biological effects via surface molecules that target recipient cells, inducing signal transduction cascades based on receptor–ligand interactions.^25^ A previous study verified that after TBI, injured neurons release EVs, which are subsequently transferred to bone and target osteoprogenitors to promote osteogenesis and accelerate bone healing.^25^ The increase in FN1 expression on the surface of sEVs contributed to direct targeting of sEVs to osteoprogenitors.^25^ In myocardial fibrosis, Ambra1 is expressed on the EV surface, and cardiac-specific Ambra1 downregulation inhibits Ambra1^+^ myocardial-sEV uptake by fibroblasts, effectively inhibiting ischemic myocardial fibrosis.^47^ Fibronectin on tumor-derived surfaces also contributes to increase in EV uptake by cancer cells.^48^ Therefore, future work will focus on the fibroblast receptor–ligand binding of BEV surface proteins by mass spectrometry and bioinformatics to further explain the specific mechanism through which BEV-targeted migration to the damaged area occurs.

In vitro, fibroblast pyroptosis increases local calcium and phosphorus concentrations and provides a microenvironment that promotes calcium deposition. Studies have revealed that vesicles derived from dying cells can concentrate calcium and act as nucleating structures for the crystallization of calcium salts.^49,50^ Calcium deposits were also found around dead cells in osteogenic culture.^51^ Furthermore, mitochondrial calcium overload is common in pyroptotic cells.^52,53^ Mitochondrial damage caused by calcium overload can act as nucleators of hydroxyapatite and form calcium nanocrystals.^54,55^ These small crystals contribute to the formation of microcalcifications.^56,57^ Our results clearly demonstrated that the formation of microcalcifications in the injured tendon occurred in the early stage of neurogenic heterotopic ossification. Local microcalcification in the injured tendon largely increases the stiffness of the extracellular matrix (ECM) around local cells, and this increase in stiffness triggers mesenchymal stem cell (MSC) osteogenic differentiation and favors the M2 macrophage phenotype.^20^ On the one hand, the Hippo signaling pathway plays an important role in the osteogenic differentiation of MSCs by reorganizing the actin cytoskeleton.^58^ On the other hand, macrophage polarization toward the M2 phenotype has been shown to be central to the formation of ectopic bone. The increase in the number of Nestin^+^ MSCs in NHO mice observed in the present study was probably due to the recruitment of circulating MSCs due to the secretion of insulin-like growth factor 1 (IGF-1) and BMPs by M2 macrophages and resulted in the formation of fully developed cancellous bone with marrow.^59^ Our results also demonstrated that the use of NSAIDs effectively inhibited fibroblast pyroptosis by suppressing the accumulation of BEVs at sites of injury, and NSAIDs were shown to be an effective medication for NHO-induced proptosis and calcium deposition.

Sympathetic and sensory neurotransmitters are indispensable for proper callus differentiation and play important roles in HO progression. Neuropeptides such as calcitonin gene-related peptide (CGRP) are released by sensory nerves and are responsible for neuroinflammation.^60–62^ CGRP is also essential for fracture healing, and after being released at the fracture site, CGRP was shown to promote the formation of type-H vessels, which couple angiogenesis and osteogenesis.^62,63^ Despite the regulatory role of the peripheral nervous system in HO, our results suggested that, unlike general heterotopic ossification, TBI accelerated heterotopic ossification in the NHO group and that the central nervous system played an important regulatory role. The sensory nervous system and sympathetic nervous system in the context of NHO should also be explored in the future. Studies comparing outcomes after mild, moderate, and severe TBI in humans have shown that deficits in cognitive domains, such as memory, attention, and information-processing speed, are increased.^64^ TBI also causes a broad range of behavioral sequelae in mice, similar to those seen in humans.^64^ Our study indicated that, similar to those in the sham group, the weights of the rats in the HO and NHO groups increased with age, and there were no significant differences among the sham, HO and NHO groups. Several myokines, such as irisin, IL-6 and muscle-derived EVs produced during activity, may also have regulatory effects on ectopic osteogenesis after TBI.^65–67^ Irisin regulates the differentiation of osteoblasts by promoting osteogenesis and antagonizing TGF-β/Smad signaling, while irisin deficiency weakens exercise-induced increases in bone strength.^65^ Skeletal muscle-derived IL-6 regulates bone remodeling during exercise, and IL-6 can boost cholinergic activity to facilitate skeletal adaptation to exercise.^66^ Muscle-derived EVs can also alter the differentiation and function of bone and muscle cells, and accumulating evidence indicates that aged skeletal muscle produces EVs that can induce senescence in stem cell populations in bone and other tissues via their miRNA cargo.^67^ Although TBI may contribute to the secretion of these myokines or EVs from muscle and have an impact on heterotopic ossification, our results suggest that BEV plays an important role in HO progression. Heterotopic calcification was more pronounced in rats injected with BEV than in those injected with PBS, and inhibiting BEV and fibroblast pyroptosis effectively reversed the occurrence of HO. These results suggest that BEV-mediated pyroptosis in fibroblasts plays a predominant role in the formation of NHO. Future research on the relationships between the formation of ectopic bone, TBI-induced changes in physical activity and several myokines is warranted.

In summary, the release of BEVs after traumatic brain injury results in the accumulation of these vesicles in the injured tendon and subsequent fibroblast pyroptosis. Pyroptosis mediates for extracellular calcium deposition and promotes NHO. Intravenous injection of a pyroptosis inhibitor could reverse the development of NHO, which provided a strategy for the prevention and treatment of NHO.

## Materials and methods

### Rat models

Male Sprague‒Dawley rats (6 weeks old, weighing 150–170 g) were used in the experiments. After being anesthetized with isoflurane, the rats were peritoneally injected with sodium pentobarbital (40 mg/kg). To construct the rat model of achillotenotomy, the rats were deeply anesthetized and then underwent bilateral midpoint achillotenotomy through a posterior approach under aseptic conditions. To construct the controlled cortical impact (CCI) rat model, the anesthetized rats were positioned in a stereotaxic frame. Then, we cut the skin of the head, performed a craniotomy using a drill (4 mm in diameter) over the right parietal-temporal cortex, exposed the dura, induced moderate brain injury by using an eCCI (electronic craniocerebral trauma instrument) and set the impact tip at a velocity of 3.0 m/s and 3.0 mm depth to hit the cerebral cortex. All incisions were closed with interrupted 4-0 silk sutures. Rats in the sham group underwent the same skin incision and craniotomy but not achillotenotomy or CCI. Rats in the HO group underwent achillotenotomy and craniotomy but not CCI. Rats in TBI group underwent achillotenotomy and CCI. All rats received analgesics to relieve pain after surgery and were allowed to move freely in their cages.

### Micro-CT

After the rats were euthanized, the crus was fixed in 4% paraformaldehyde and analyzed on a Micro-CT scanner (Inveon Micro-CT system, Siemens AG, Germany; 80 keV and 500 mA; 10 μm isotropic). We calculated the length of the tendon as the distance between the heel bone (tendon-bone junction) and the muscle (tendon-muscle junction, the sudden enlargement of the tendon). Then, the tendon was selected by threshold segmentation of the value used for the soft tissue. Total tendon volume was calculated as the sum of the gray pixels in the posterior tibial region.^68^ Three-dimensional reconstruction and morphometry were performed to calculate bone microarchitectural parameters, including bone mineral density (BMD), bone volume (BV), bone trabecular thickness (Tb.Th) and trabecular number (Tb.N).

### Histological evaluation

After fixation, the tissues were decalcified with 10% EDTA (6381-92-6; Solarbio, CN) solution with gentle shaking for 1 month. After decalcification, the samples were embedded in paraffin and cut into 6-µm-thick sections. After being dewaxed and hydrated, the sections were stained with hematoxylin-eosin (G1120, Solarbio, CN) and Safranin O and Fast Green (S8884, Sigma-Aldrich) and observed by microscopy (DM4, Leica, Germany). Bone trabecular thickness (Tb.Th) and trabecular number (Tb.N) were calculated by ImageJ software (National Institute of Health, Bethesda, USA). The sections were stained with alizarin red S (40 mmol/L, pH = 4.2; MilliporeSigma, Burlington, USA) for 20 min. The nuclei were counterstained with DAPI (Invitrogen). The relative fluorescence intensity was analyzed by ImageJ software (National Institute of Health, USA).

### Immunohistochemistry

Before immunohistochemical and immunofluorescence staining, the paraffin-embedded sections were dewaxed, hydrated and subjected to heat-induced epitope retrieval. For cell samples, we used 4% paraformaldehyde to fix the cells for further staining. For immunohistochemical staining, we diluted the following primary antibodies in QuickBlock™ Primary Antibody Dilution Buffer (P0262; Beyotime, CN): NLRP3 (DF15549; Affinity, USA; 1:200 dilution) and CASPASE-1 p20 (AF4005; Affinity, USA; 1:200 dilution). Hydrogen peroxide (0.3%) was used to block endogenous peroxidase activity. After being blocked with goat serum (C0265, Beyotime, CN) and incubated with the primary antibody at 4 °C overnight, the sections were washed with PBS (P1020, Beyotime, CN), followed by incubation with the anti-rabbit IgG secondary antibody (Kit-5010, MXB, CN) for 1 h at 37 °C. Color was developed with DAB solution (DAB-4033, MXB, CN), and the sections were counterstained with hematoxylin. For immunofluorescence analysis, we diluted primary antibodies against alpha-SMA (BF9212, Affinity, USA; 1:300 dilution), N-GSDMD (DF13758, Affinity, USA; 1:300 dilution), L1CAM (bs-1996R, Bioss, CN; 1:300 dilution), CD63 (67605-1-Ig, Proteintech, USA; 1:300 dilution), F4/80 (DF2789, Affinity, USA; 1:300 dilution), CD4 (DF16080, Affinity, USA; 1:300 dilution), and Ly6G (orb322983, Biorbyt, CN; 1:300 dilution). For secondary reactions, we used species-matched Alexa Fluor 488 AffiniPure Donkey Anti-Mouse IgG (H+L) (PC-80014; PlantChemMed Biology Co., Ltd., ShangHai, China) and Alexa Fluor 594 AffiniPure Donkey Anti-Rabbit IgG (H+L) (PC-80009; PlantChemMed Biology Co.) antibodies at 37 °C in the dark. The sections were mounted with prolonged antifade mountant containing 4′,6-diamidino-2-phenylindole (DAPI) (S2110; Solarbio, CN). TUNEL staining (C1091, Beyotime, CN) and Hoechst/PI staining (CA1120, Beyotime, CN) were performed according to the protocols provided by the manufacturers. We observed the sections with a confocal microscope (FV1000, Olympus, Japan). Finally, the data were analyzed with ImageJ software (National Institute of Health, USA). For all the immunohistochemical and immunofluorescence staining experiments, there were three biological replicates.

### Transmission electron microscopy (TEM)

Glutaraldehyde (2.5%) in PBS was used to fix the tendons (0.01 mol/L, pH = 7.4). The specimens were then fixed in 1% osmium tetroxide for 1 h and dehydrated in an ascending series of ethanol. The dehydrated specimens were immersed in propylene oxide and embedded in epoxy resin. After being dissected and prepared, 90-nm-thick sections were cut and stained with uranyl acetate and lead citrate. A JEM-123 transmission electron microscope (TEM, JEOL, Tokyo, Japan) was used to observe the sections at 110 kV.

### Flow cytometry

After isolating EVs from the plasma of HO rats and NHO rats, 200 μL of PBS was used to resuspend each kind of EV. Then, these EVs were incubated with an anti-L1CAM antibody (diluted 1:150; ab272733; Abcam, USA) for 30 min. After the mixture was centrifuged at 100 000 r/min for 1 h, the EVs were resuspended in PBS. The EVs were incubated with secondary antibodies (diluted 1:300; SA00014-9; Proteintech, USA) after the addition of 2% serum albumin. After total EVs were washed with 2% bovine serum albumin in PBS, L1CAM^+^ BEVs were detected by a flow cytometer (CytoFLEX, Beckman Coulter, California, USA).

### Scanning electron microscopy (SEM) and energy-dispersive X-ray spectroscopy

Glutaraldehyde (2.5%) in phosphate buffer (0.01 mol/L, pH = 7.4) was used to fix the tendons. The specimens were then dehydrated with an ascending series of ethanol and treated with hexamethyldisilane (Electron Microscopy Sciences, Hatfield, PA, USA). A field-emission scanning electron microscope (FE-SEM, S-4800, Hitachi, Tokyo, Japan) operating at 5 kV was used to observe the samples. The mineral elemental composition of the tendons was characterized by using energy-dispersive X-ray spectroscopy (Element EDS System, Ametek, Berwyn, PA, USA).

### Plasma BEVs isolation

After the rats were anesthetized, peripheral blood was collected in tubes containing EDTA as an anticoagulant. We centrifuged the samples (1 200 × *g*, 10 min, 4 °C) immediately to separate the plasma from the peripheral blood. The plasma was carefully collected and transferred to a new tube without disturbing the intermediate buffy coat layer. After the samples were incubated for 10 min at room temperature, the plasma was centrifuged (3 000 × *g*, 20 min, 4 °C), and the supernatant was collected after an additional centrifugation step (11 000 × *g*, 30 min, 4 °C). The supernatant was subsequently transferred to a new tube and centrifuged at 18 000 × *g* for another 30 min at 4 °C. Finally, we collected the supernatant in a new tube and centrifuged it at 100 000 × *g* for 2 h at 4 °C to precipitate total EVs in the plasma. Total plasma EVs were resuspended in 200 μL of PBS with inhibitor cocktails, and 100 µL of L1CAM biotinylated antibody (mouse anti-rat) (bs-1996R-Bio, Bioss, Bioss, CN) in 50 µL of 3% BSA (bovine serum albumin) was added and mixed. The mixture was incubated for 1 h at 4 °C. After that, we added 10 μL of streptavidin-agarose UltraLink Resin (20512ES08, Yeasen, CN) and 40 µL of 3% BSA, followed by incubation for 30 min at 4 °C. After centrifugation (400 × *g*, 15 min, 4 °C), each pellet of agarose settled to the bottom of the tube, and BEVs were adsorbed on the surface of the pellets. The supernatant contained BEVs-depleted EVs. Each agarose pellet was resuspended in 100 µL of 0.05 mol/L glycine-HCl (pH 1.0) to release the BEVs from the surface of the pellets. Then, the mixture was centrifuged at 4 000 × *g* for 10 min at 4 °C. The final supernatant contained BEVs. Finally, we added cocktails of protease and phosphatase inhibitors (78440; Thermo Fisher Scientific, USA) at the recommended concentrations to each tube, and the suspensions were stored at –80 °C until further use.

### Brain BEVs isolation

We dissected and separated rat brains after they were sacrificed. Five hundred milligrams of brain tissue was cut and added to culture medium supplemented with lyophilized papain (P4762; Sigma, USA; 20 U/mL). The brain tissue was crushed and incubated at 35 °C for half an hour. Then, the mixture was centrifuged, and the BEVs were separated from the obtained supernatant according to the aforementioned separation steps.

### Characterization of BEVs

A total of 10 μL of each BEV suspension was placed on a copper mesh and incubated for 20 min at room temperature. Then, the copper mesh was dried by placing it sideways on filter paper, after which it was immersed in saturated uranyl acetate for 3 min and immersed in pure water for 5 min. The samples were subsequently dried and observed by transmission electron microscopy (TEM; JEOL, Tokyo, Japan). Prior to analyzing particle size distribution, the samples were diluted in PBS to obtain a range of 60%-70% transmittance and then analyzed by NanoSight NTA Software (Litesizer500, Anton Paar, GER). The concentration of BEVs was then determined by a BCA protein assay (PC0020, Solarbio, CN) according to the manufacturer’s instructions. We performed Western blot analysis according to standard protocols. The EVs were subjected to lysis buffer (P0013F; Beyotime, CN), separated by SDS‒polyacrylamide gel electrophoresis (#1610183; Bio-Rad Laboratories, UK) with running buffer (B0002; Thermo Fisher Scientific, USA), and transferred to polyvinylidene fluoride (PVDF) membranes (IEVH85R; Merck, USA) in an ice bath. The membrane was blocked with 5% BSA for 2 h and then incubated overnight with primary antibodies at 4 °C. Primary antibodies against TSG101 (Ab125011, Abcam, USA, 1:1 000 dilution), CD9 (Ab92726, Abcam, USA, 1:1 000 dilution), CD63 (Sc-5275, Santa Cruz, Thermo Fisher Scientific, USA, 1:1 000 dilution), L1CAM (BS-1996R, Bioss, CN, 1:1 000 dilution) and calnexin (10427-2-AP, Proteintech, USA, 1:1 000 dilution) were used. The secondary antibodies used for western blotting were anti-rabbit IgG (cat# 7074; Cell Signaling Technology, USA; dilution, 1:2 000 dilution) and anti-mouse IgG (cat# A9044; Sigma-Aldrich, USA; dilution, 1:2 000 dilution). Finally, the membrane was incubated with an enhanced chemiluminescence HRP substrate (WBKLS0100, Millipore, USA) for 3 min and observed with an imaging system (Azure 600, Azure Biosystems, CN).

### Cell culture

L929 cells (a mouse fibroblast line) purchased from Procell were seeded in DMEM (PM150421, Pricella, CN) supplemented with 10% fetal bovine serum (PC-00001; PlantChemMed Biology Co.) and 1% penicillin streptomycin solution (100X) (PC-86115; PlantChemMed Biology Co.) in 5% CO_2_ at 37 °C.

### Exosome labeling and cellular uptake assay

BEVs were labeled with the red fluorescent agent PKH26 (PKH26GL-1KT; Sigma-Aldrich, USA) and centrifuged at 100 000 × *g* for 70 min to remove unbound dye. Then, the BEVs were suspended in PBS. We cocultured PKH26-labeled BEVs (500 μg/dish) with L929 cells. At the indicated times, we stained the cytoskeleton with phalloidine (49409-10NMOL; Sigma-Aldrich, USA; 5 μg/mL) and observed the cells by confocal microscopy (FV1000; Olympus, Tokyo, Japan). After being cultured for 3 days, the cells were stained with N-GSDMD and Hoechst 33342/PI according to the instructions. To further assess the effect of BEVs on pyroptosis and calcification, we added EVs (500 μg per dish), BEVs-depleted EVs (500 μg per dish) and BEVs (500 μg per dish) combined with a Caspase-1 inhibitor (Ac-YVAD-cmk, HY-16990, MedChemExpress, USA; 1 mg/mL dilution) to the cell culture system for 3 or 7 days. We assessed pyroptosis in the coculture system by performing green immunofluorescence staining for N-GSDMD after 3 days. To assess the effect of the BEVs on calcification, the system was maintained for 7 days. After being fixed with 4% paraformaldehyde, the cells were stained with Alizarin Red S (50 mmol/L, pH = 7.2). We removed the excess dye by washing the samples with water for 30 min. A Zeiss microscope (Thorn-wood, NY, USA) was used to image the plates. The stained areas were measured with ImageJ software.

### Western blot analysis of fibroblast pyroptosis in response to BEVs

After fibroblasts were cocultured with BEVs, we lysed the cells in RIPA buffer (P0013B, Beyotime, CN). We separated the proteins by SDS‒polyacrylamide gel electrophoresis (#1610183; Bio-Rad Laboratories, UK) and blocked the samples with BSA. After the proteins were blotted onto polyvinylidene fluoride (PVDF) membranes, the membranes were incubated with N-GSDMD (#DF12275; Affinity, USA; 1:300 dilution), HMGB1 (#AF7020; Affinity, USA; 1:300 dilution) and β-actin (#4970S; Cell Signaling Technology, USA; 1:1 000 dilution). The secondary antibodies used for western blotting were anti-rabbit IgG (cat# 7074; Cell Signaling Technology, USA; dilution 1:2 000) and anti-mouse IgG (cat# A9044; Sigma-Aldrich, USA; dilution 1:2 000 dilution). An enhanced chemiluminescence HRP substrate (WBKLS0100, Millipore, USA) was used to visualize and analyze the membrane.

### Mass spectrometry analysis of BEVs

BEVs were isolated and treated with RIPA lysis buffer. After trypsin digestion, the mixture was injected into a mass spectrometer (Orbitrap Eclipse Tribrid Mass Spectrometer, Thermo Fisher Scientific, USA). After calibrating the system with the standard compounds, the mass spectrometer was operated in the data-dependent mode. In this mode, the mass spectrometer cycled between full MS scans with m/z 100–1 800. Only proteins with high protein FDR confidence were considered for further analysis.

### Detection of phosphorus and calcium concentrations in the supernatant of fibroblasts

Dynamic monitoring of phosphorus and calcium concentrations in the supernatant of fibroblasts was performed at different times after being cocultured with BEVs filtered through a 0.22-mm filter. Detection was performed by inductively coupled plasma‒optical emission spectrometry (ICP–OES, Agilent 5900, Agilent, USA). The analysis was repeated three times under the same conditions.

### Fluorescence imaging analysis of BEV distribution

To track BEVs in vivo, we injected PKH26-labeled BEVs into rats that had undergone achillotenotomy. One hundred microliters (intravenously, 6 µg/µL) of BEVs were injected into each rat. Then, the rats were killed 3 days after the injection, and the tendons were harvested. The specimens were fixed in 4% paraformaldehyde for 24 h and 30% sucrose for 3 days. After that, optimal cutting temperature compound (Leica, Wetzlar, Germany) was used to embed the specimens. Then, the specimens were stored at –80 °C. Five-micron-thick sections were cut from the specimens and stored for further experiments. The BEVs in the Achilles tendon were imaged by confocal microscopy (FV1000, Olympus, Tokyo, Japan).

### GW4869 administration

To reduce the concentration of BEVs in plasma, we used GW4869 (D1692; Sigma-Aldrich, USA), which is a compound that can inhibit the secretion of BEVs from the central nervous system. GW4869 was diluted to a final concentration of 0.25 mg/mL in 0.9% normal saline and then intravenously injected into rats (250 µg/100 g body weight, twice per week).

### Ac-YVAD-cmk Injections

To ameliorate pyroptosis in fibroblasts in the Achilles tendon after TBI, Ac-YVAD-cmk (178603-78-6; Sigma-Aldrich, USA) was dissolved in normal saline and injected into rats at a dose of 1 μg/rat three times per week. An equal volume of normal saline was injected into the mice in the sham group.

### Indomethacin administration

To explore the inhibitory effects of nonsteroidal anti-inflammatory drugs on NHO, indomethacin, which is a common clinical nonsteroidal anti-inflammatory drug, was administered to rats in the NHO group. Indomethacin (CAS:53-86-1; Solarbio, Beijing, China) was diluted to a concentration of 3 mg/kg, after which the rats in the NHO group were orally administered indomethacin by syringe feeding after surgery.

### Parallel experiment

A traumatic brain injury rat model was constructed in which the Achilles tendons of the left leg was sham-treated (sham) and those of the right leg were injured (injured). All rats received analgesics to relieve pain after surgery and were sacrificed 3 days postinjury for immunofluorescence staining and 3 weeks postinjury for micro-CT and H&E staining.

### Statistical analysis

Analyses were performed using GraphPad Prism 8.0 (GraphPad Software, USA). All the data are presented as the means ± standard deviations. The Shapiro‒Wilk test and modified Leven test were used to test the normality and homoscedasticity assumptions of the corresponding datasets, respectively. Student’s *t*-test and one-factor or two-factor analysis of variance followed by Holm–Šidák multiple comparison tests were used to evaluate the differences among groups. For all tests, statistical significance was set at *α* = 0.05.

### Supplementary information

Supplementary Information

Supplementary Table 1

## Data Availability

The mass spectrometry proteomic data have been deposited in the ProteomeXchange Consortium via the iProX partner repository with the dataset identifier PXD046626.
