# Supplementary material for: Interorgan communication in neurogenic heterotopic ossification: the role of brain-derived extracellular vesicles
Source: Bone Res. 2024 Feb 22;12:11. doi: 10.1038/s41413-023-00310-8 (PMC10881583; doi:10.1038/s41413-023-00310-8)
Supplement: Supplementary file 1 — Supplementary Information [file 41413_2023_310_MOESM1_ESM.docx]

**Interorgan communication in the neurogenic heterotopic ossification: focusing on the role of brain-derived extracellular vesicles**

**Supplementary Information**

**
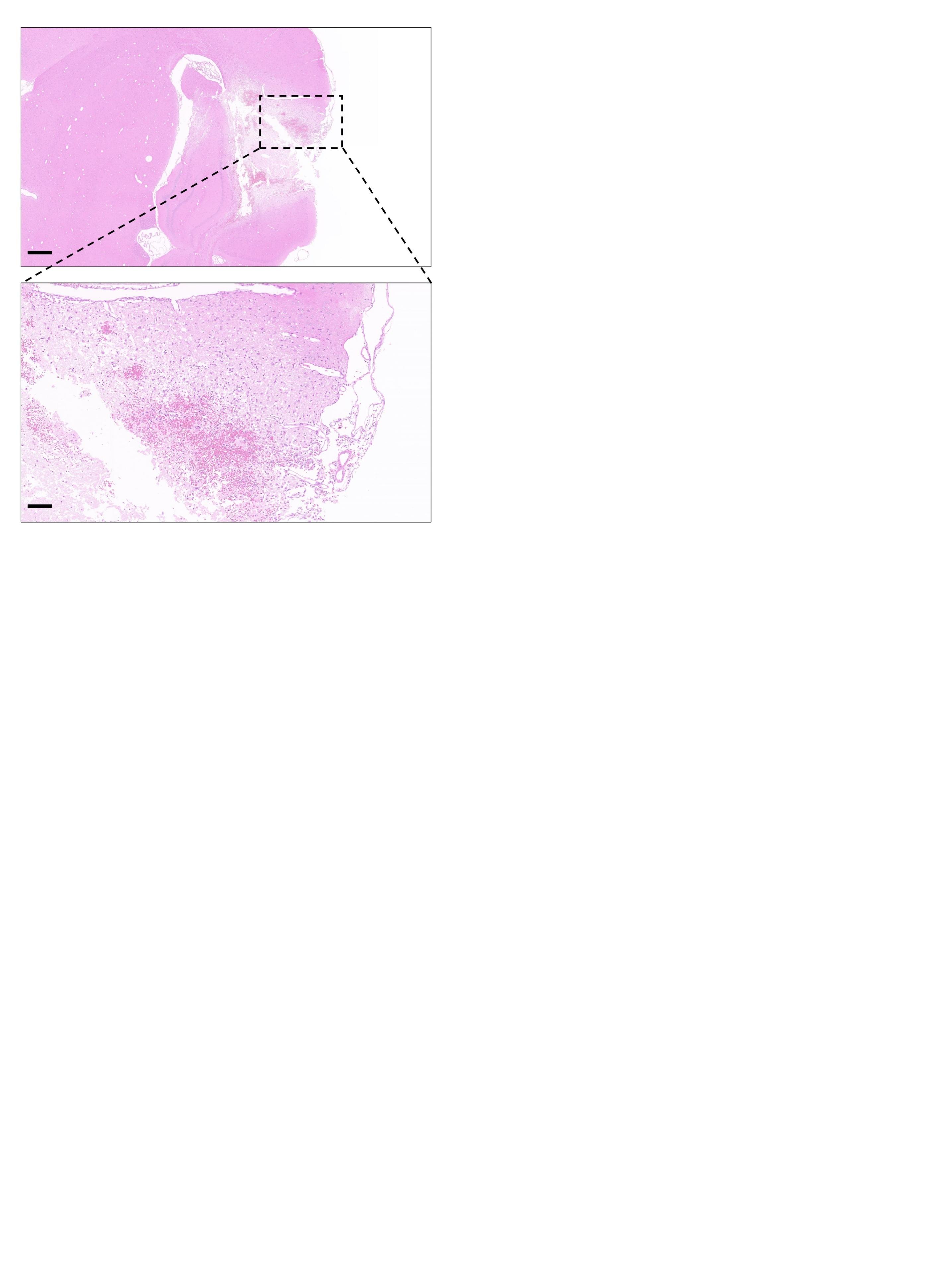
**

**Fig. S1.** H&E staining of the brain area affected by the controlled cortical impact (CCI) rat model. Scale bar, 500 μm (upper), 100 μm (lower).

**
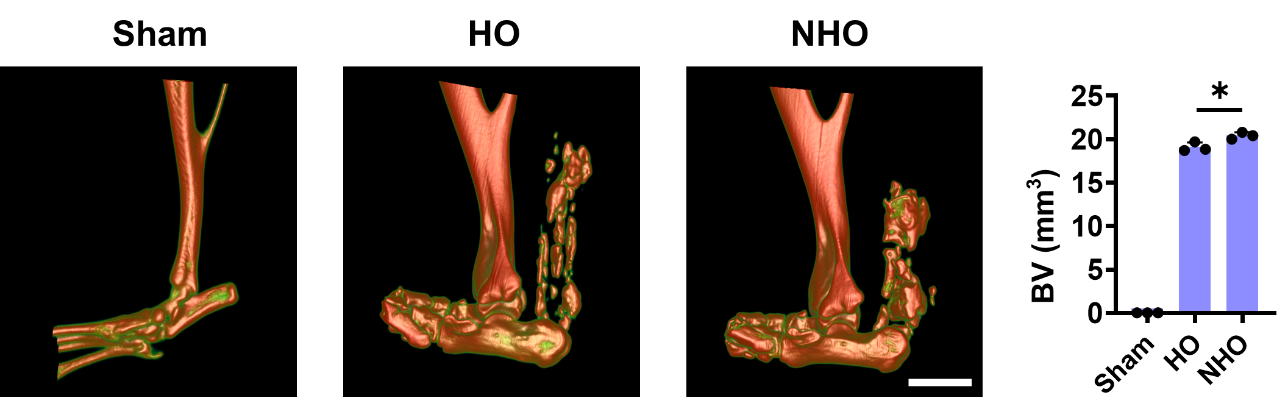
**

**Fig. S2.** Micro-computed tomography (micro-CT) images of Achilles tendon of rats from the sham, HO and NHO groups after 12 weeks. Scale bar, 5 mm. Data represent means ± standard deviations (n = 3). Statistical analyses were performed using one-way ANOVA with post-hoc Tukey’s test. *P < 0.05.

**
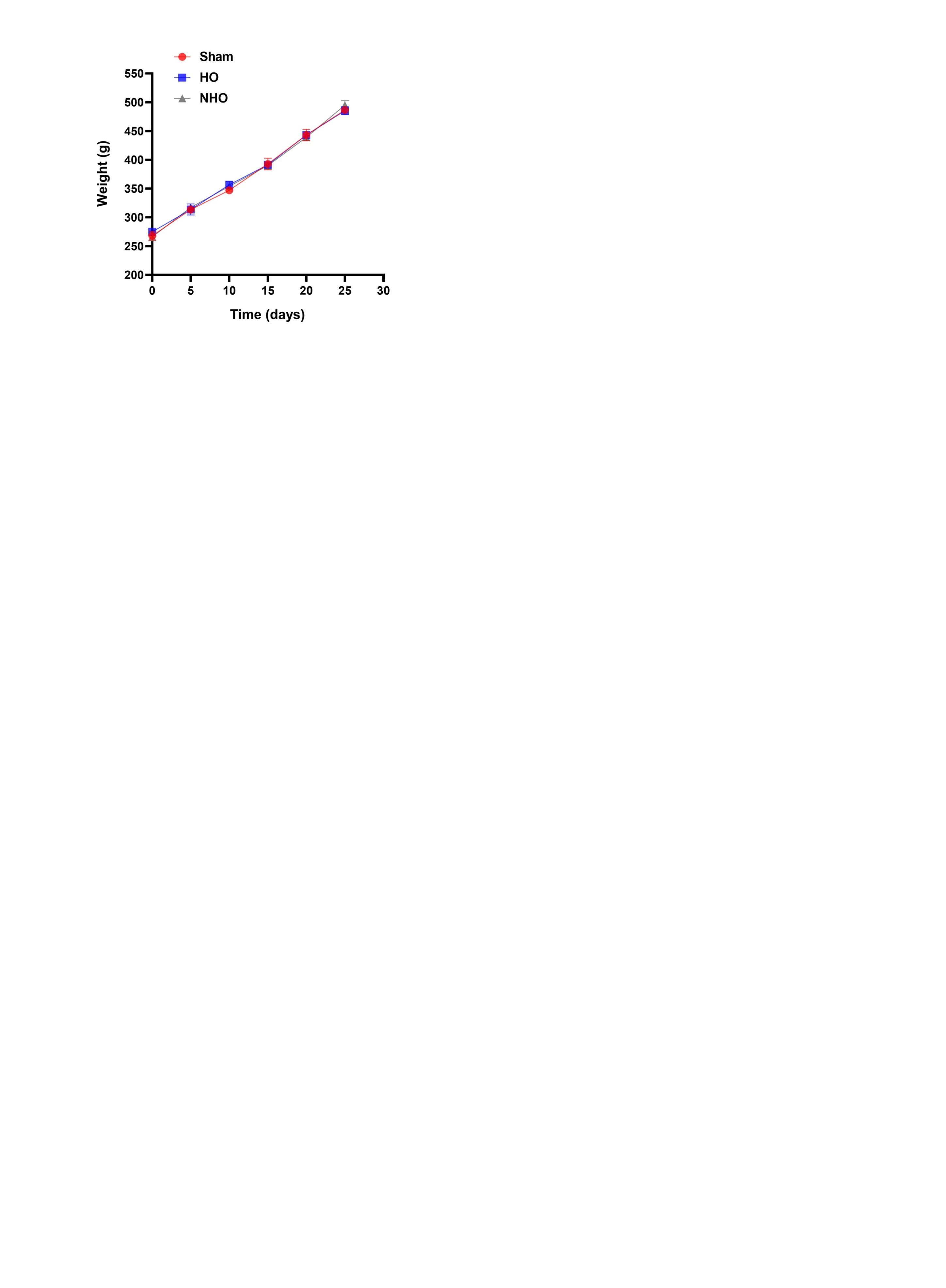
**

**Fig. S3.** Trend of weight changes in rats with different treatments from 0 to 25 days. Data were presented as means ± standard deviations (n = 3). Statistical analyses were performed using two-way ANOVA with post-hoc Tukey’s test.

**
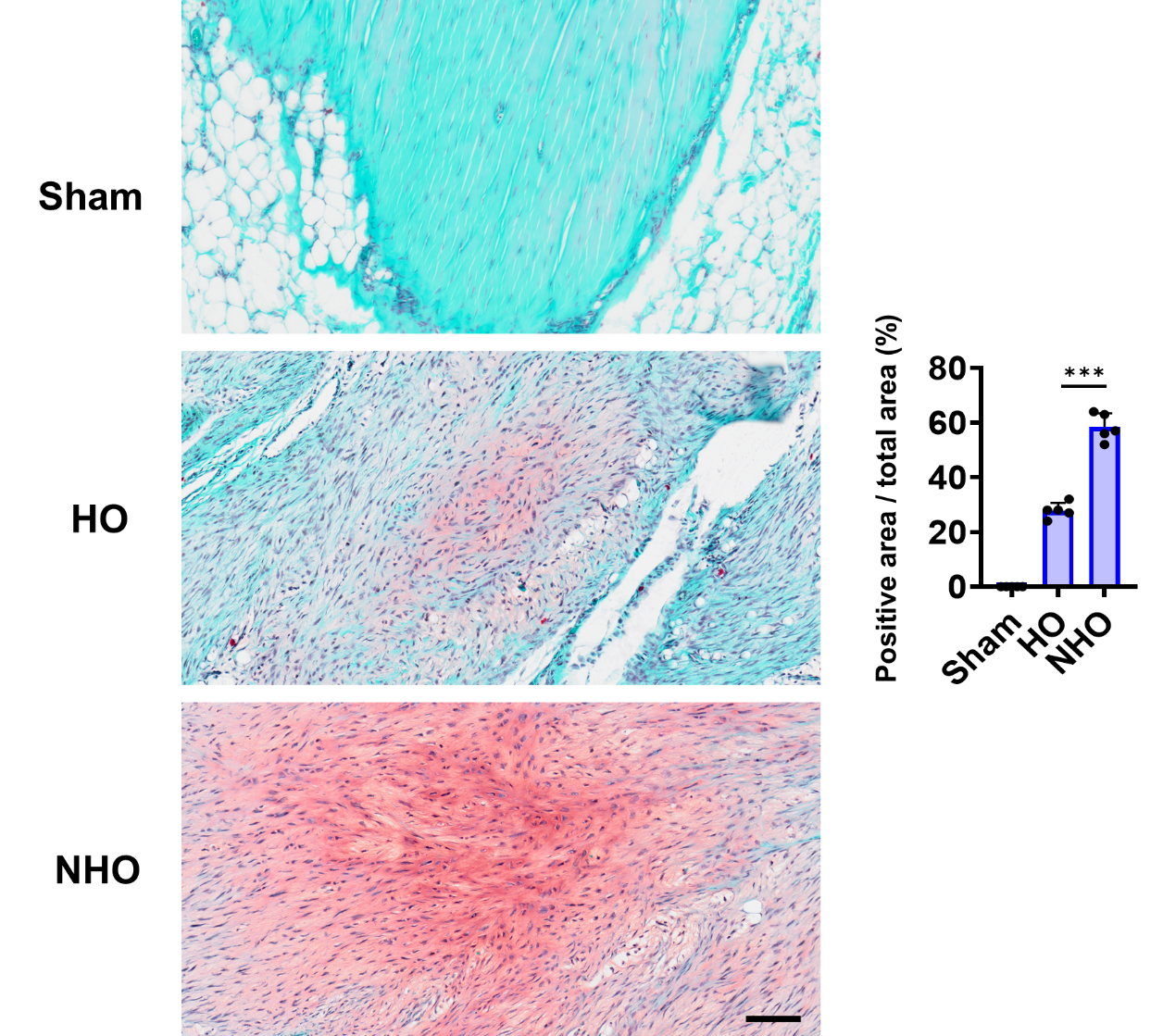
**

**Fig. S4.** Safranin O and Fast Green (SOFG) staining of the tendon and quantification. Proteoglycan (red). Scale bar, 100 μm. Data were presented as means ± standard deviations (n = 5). Statistical analyses were performed using one-way ANOVA with post-hoc Tukey’s test. ***P < 0.001.


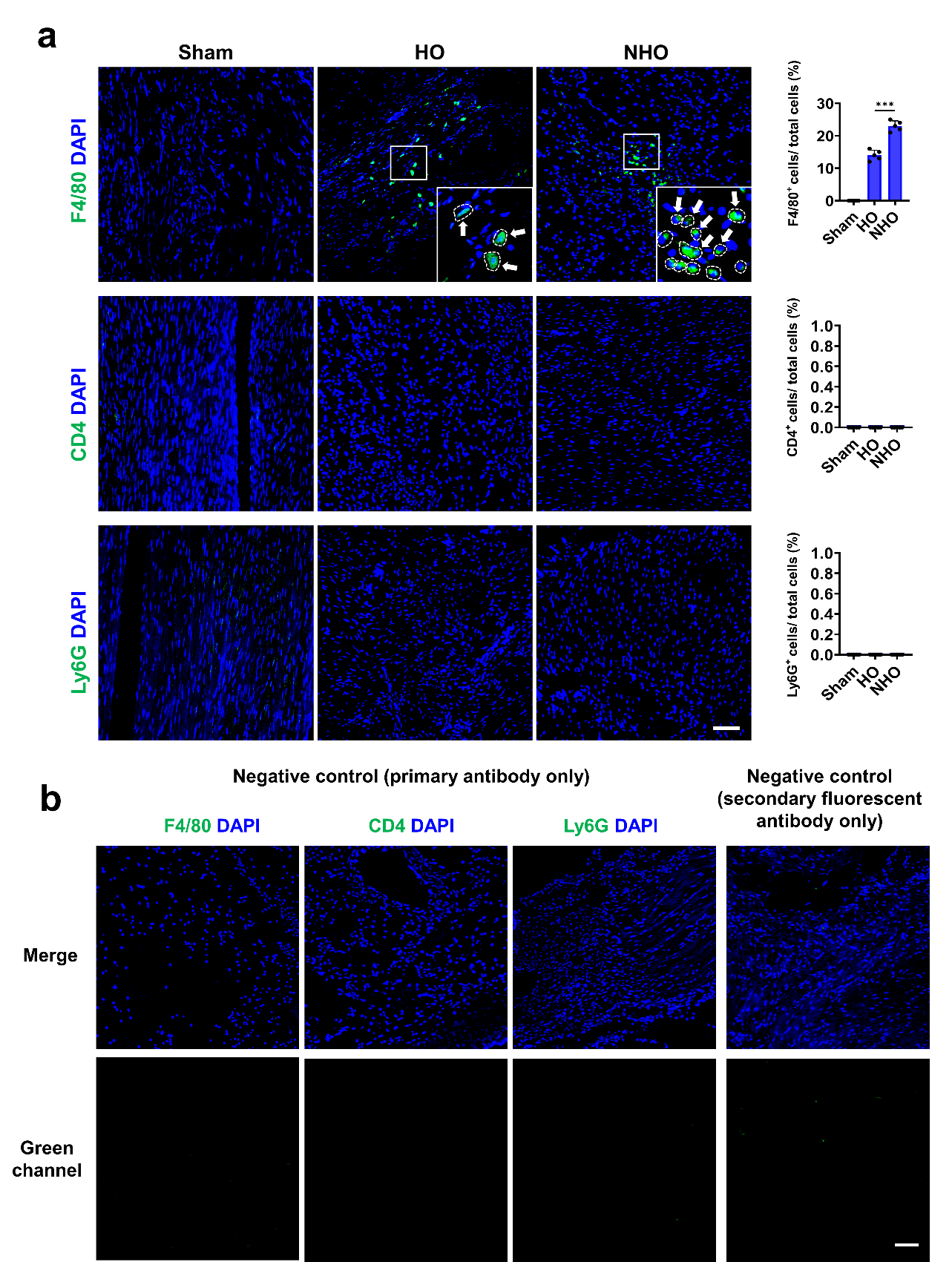


**Fig. S5.** **(a)** F4/80^+^ (macrophage marker (arrows)), CD4^+^ (T cell marker), Ly6G^+^ (neutrophil marker) (green) cells in the tendon and quantification. Scale bar, 50 μm. Blue indicates DAPI staining of nuclei. Data were presented as means ± standard deviations (n = 5). Statistical analyses were performed using one-way ANOVA with post-hoc Tukey’s test. ***P < 0.001. **(b)** Controls for confocal imaging for a. Scale bar, 50 μm.

**
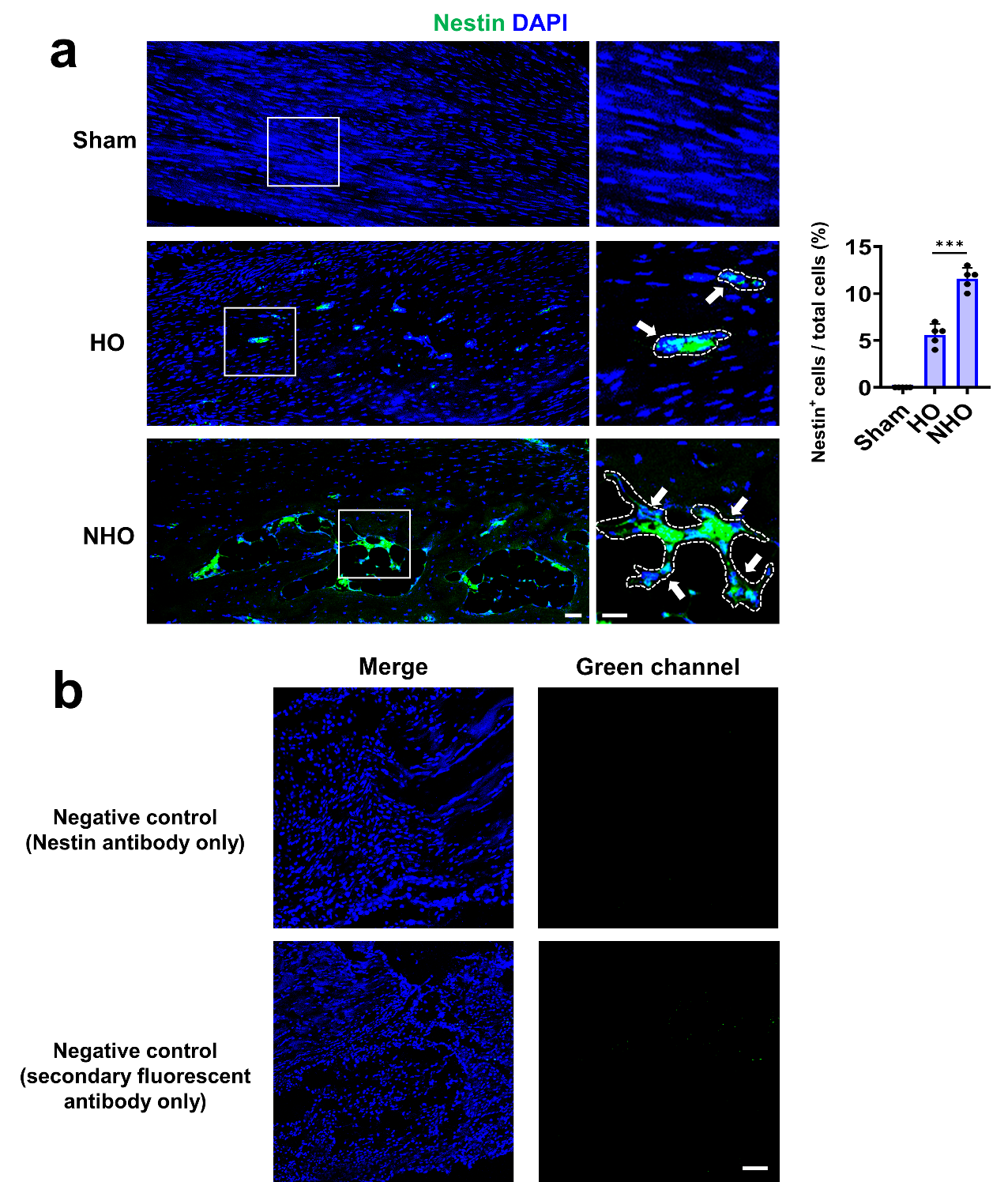
**

**Fig****. S6. (a)** Nestin^+^ (green, arrows) cells in the tendon and quantification. Scale bar, 30 μm (left); 10 μm (right). Blue indicates DAPI staining of nuclei. Data were presented as means ± standard deviations (n = 5). Statistical analyses were performed using one-way ANOVA with post-hoc Tukey’s test. ***P < 0.001. **(b)** Controls for confocal imaging for a. Scale bar, 50 μm.

**
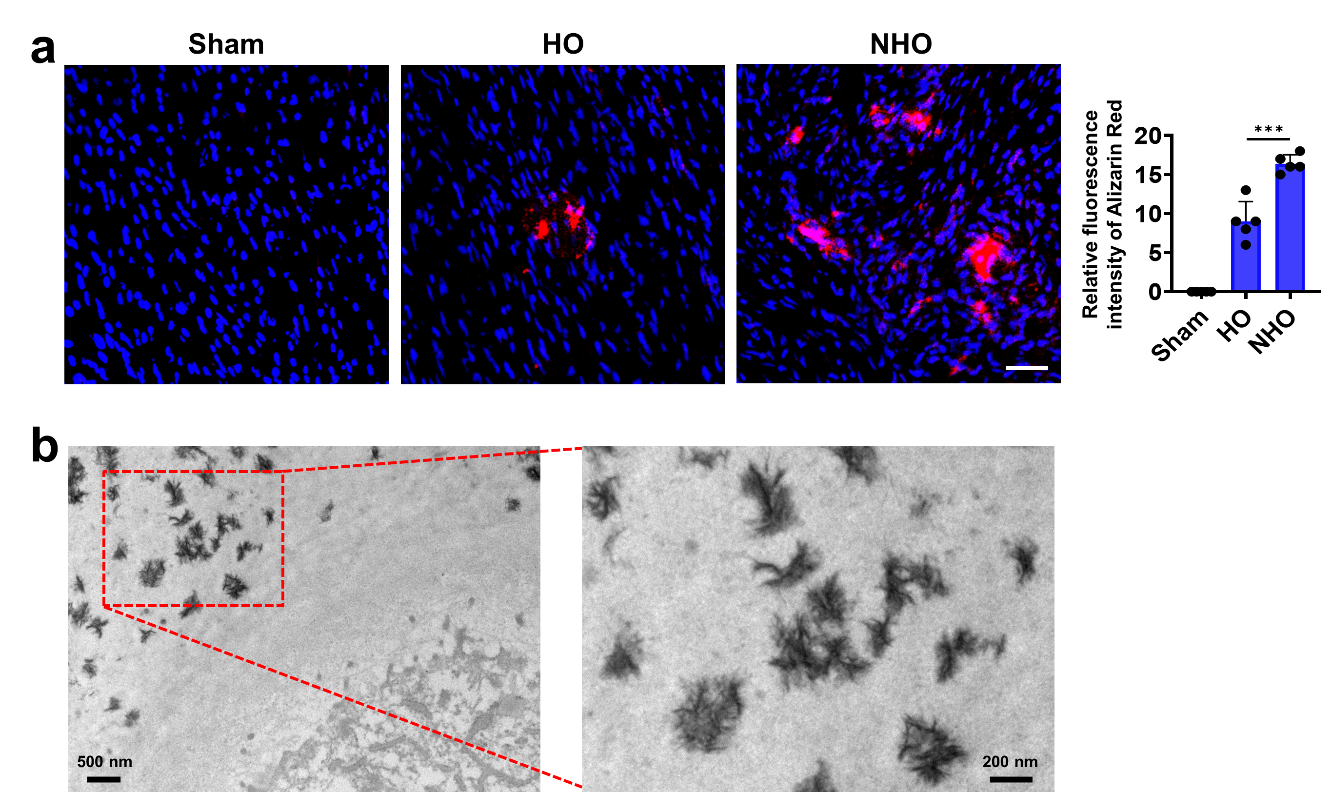
**

**Fig. S7. (a)** Alizarin red S staining of Achilles tendons of rats from the sham, HO and NHO groups after 1 week. Areas stained with alizarin red S indicated calcified regions. Alizarin red S, red; DAPI, blue. Scale bar: 50 μm. Quantitative analysis of the calcified regions stained with Alizarin red S was performed. Data were presented as means ± standard deviations (n = 5). Statistical analyses were performed using one-way ANOVA with post-hoc Tukey’s test. ***P < 0.001. **(b)** Representative TEM images of the Achilles tendons of rats from the NHO groups after 1 week.

**
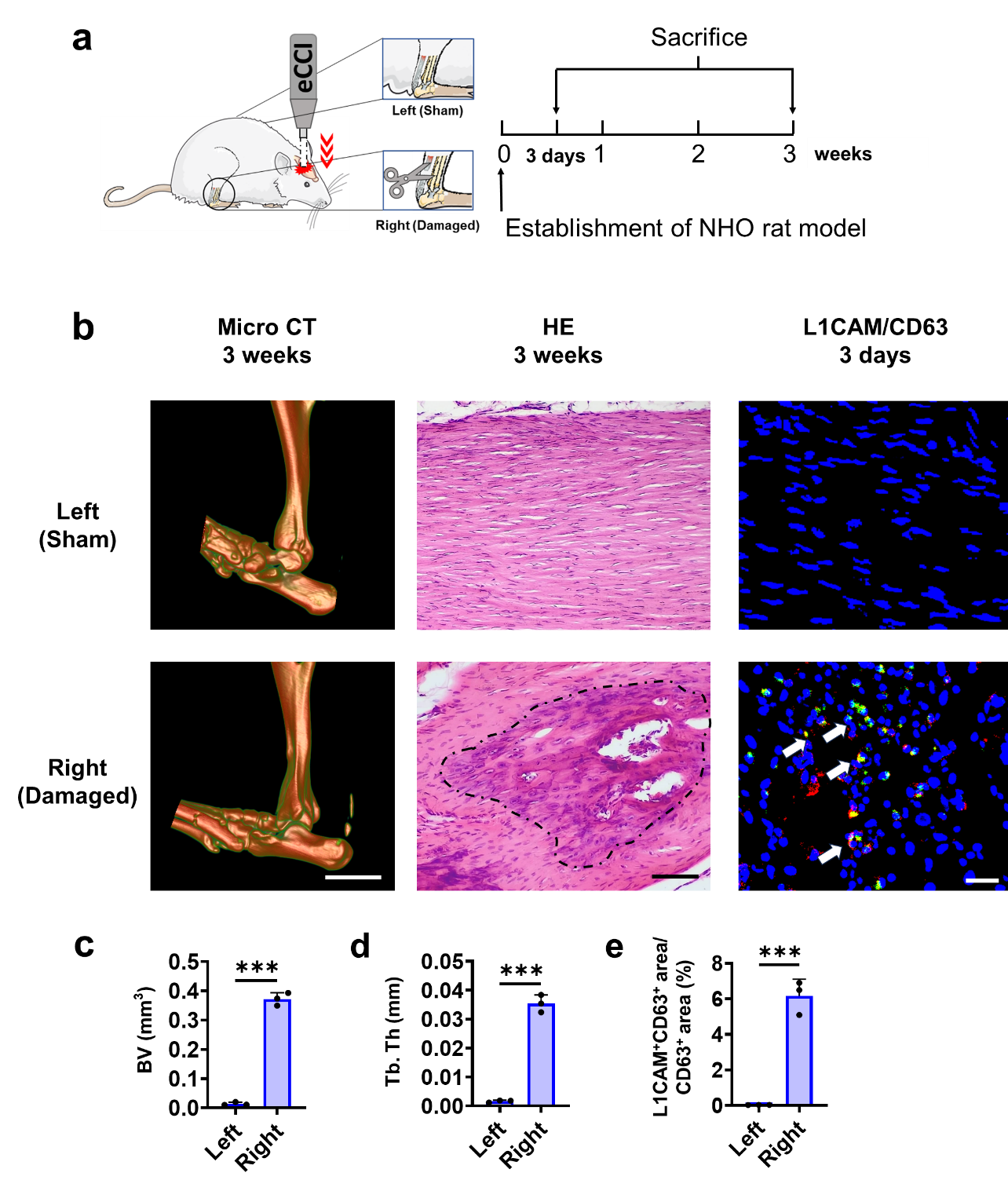
**

**Fig. S8. (a)** Schematic representation of NHO rats with the left tendon (untreated) and the right tendon (treated). **(b)** Micro-CT images (scale bars, 5 mm), H&E staining (scale bars, 100 μm) and immunofluorescence staining (L1CAM, green; CD63, red; scale bars, 100 μm) images of heterotopic calcification in the left and right Achilles tendon. BEVs were indicated by arrows. Areas within the dotted black lines indicate bone trabecula. **(c-e)** Quantitative analysis of BV, Tb. Th and the percentage of L1CAM^+^ CD63^+^ area in the CD63^+^ areas from the two groups. Data were presented as means ± standard deviations (n = 3). Statistical analyses were performed using Student’s t-test. ***P < 0.001.

**
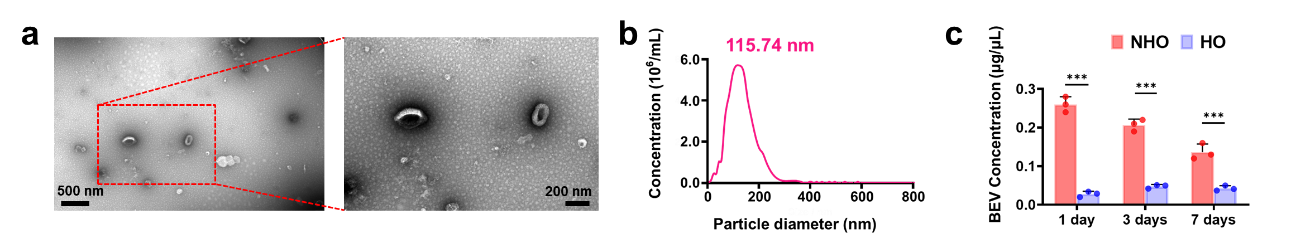
**

**Fig. S9. Characterization and isolation of BEVs in NHO.** **(a)** TEM images of the BEVs from the brains of rats from NHO groups after 3 days. Scale bar: 500 nm. High magnification of the area depicted by the red rectangle in the low magnification image. Scale bar: 200 nm. **(b)** Nanoparticle tracking analyses of the BEVs from the rat brains derived from the NHO groups after 3 days. **(c)** Quantification of BEV concentration in rats from the brains of the HO and NHO groups at 1, 3 and 7 days after injury. Data represent means ± standard deviations (n = 3). Statistical analyses are performed by two-way ANOVA with post-hoc Tukey’s test. ***P < 0.001.


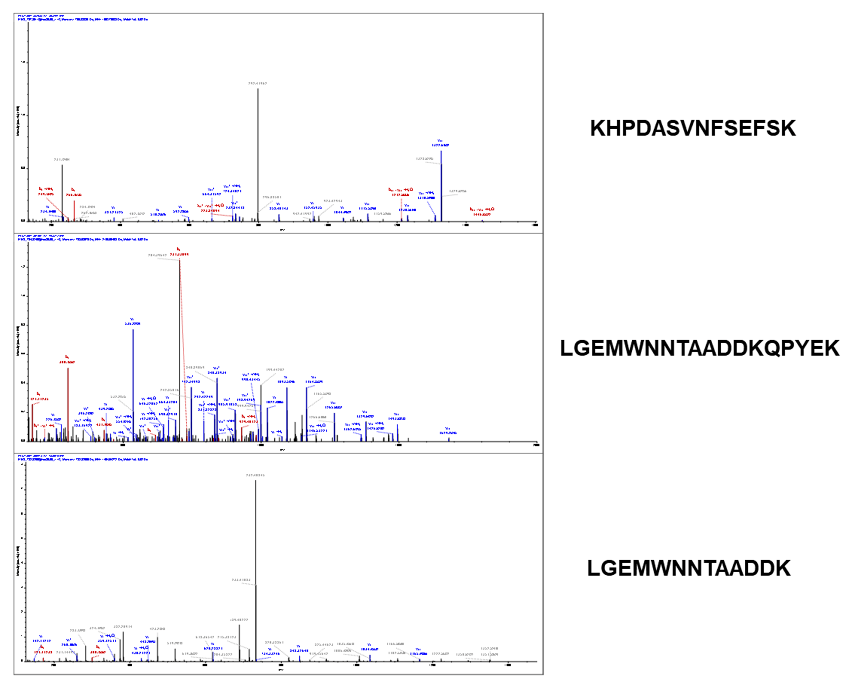

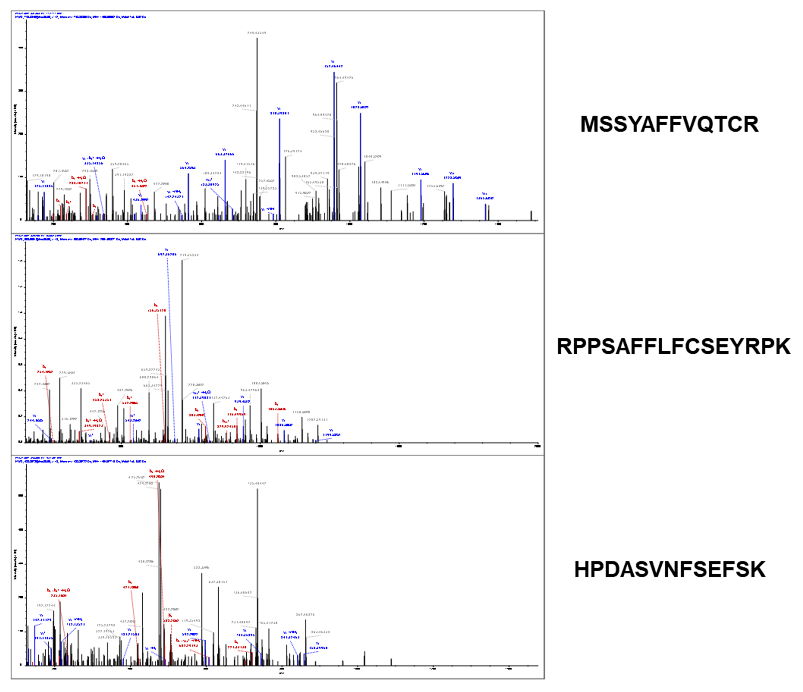


**Fig. S10.** Other 6 peptides of HMGB1 identified by the mass spectrometer.

**
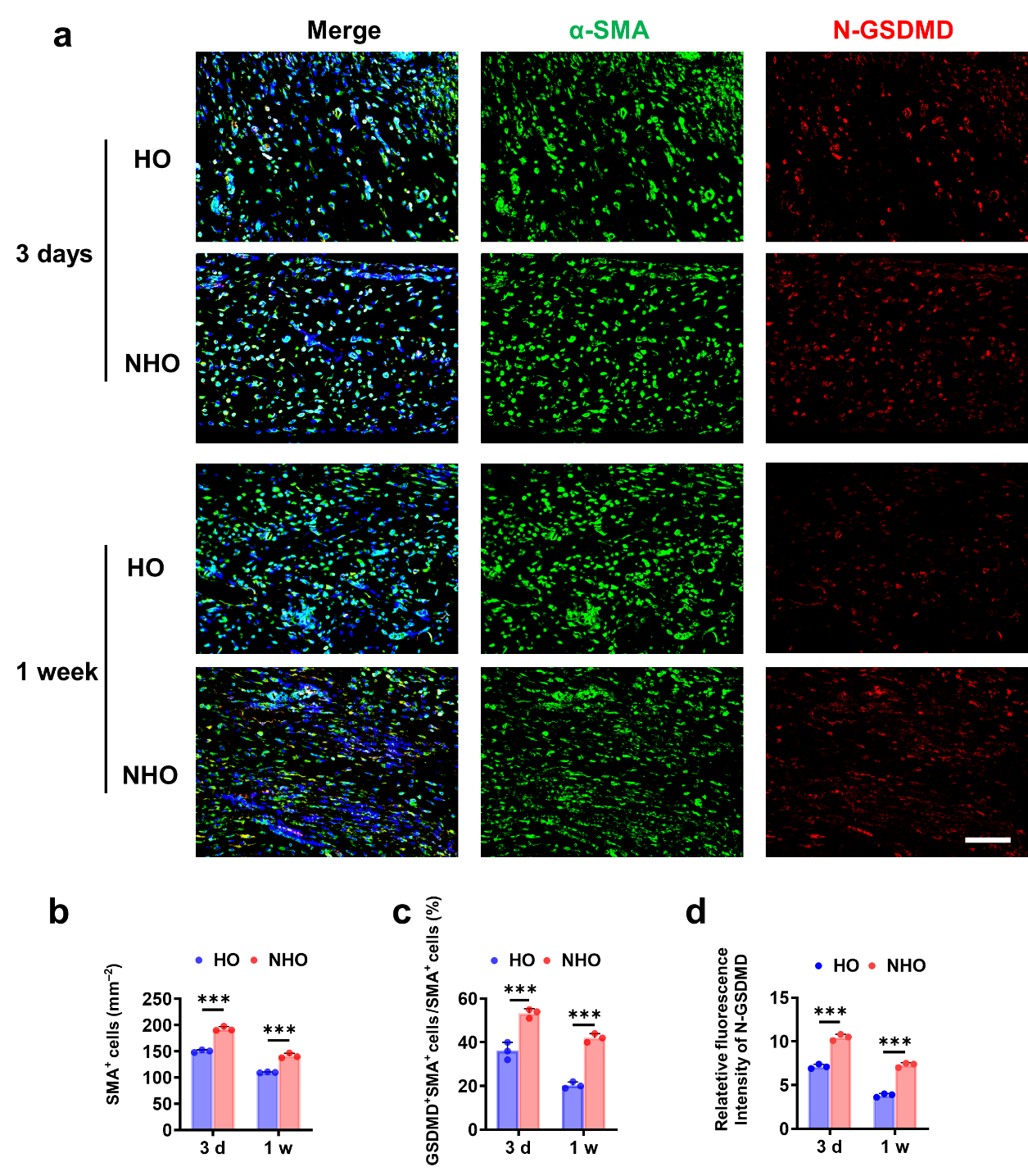
**

**Fig. S11. (a)** Representative confocal images of N-GSDMD (red), α-SMA (green) and DAPI (blue) in the rat tendons after traumatic brain injury and achillotenotomy. Scale bar, 50 μm. **(b, c, d)** Quantitative analysis of SMA^+^ cells, GSDMD^+^ SMA^+^ cells /SMA^+^ cells and relative fluorescence Intensity of N-GSDMD. Data were presented as means ± standard deviations (n = 3). Statistical analyses were performed using two-way ANOVA with post-hoc Tukey’s test. ***P < 0.001.

**
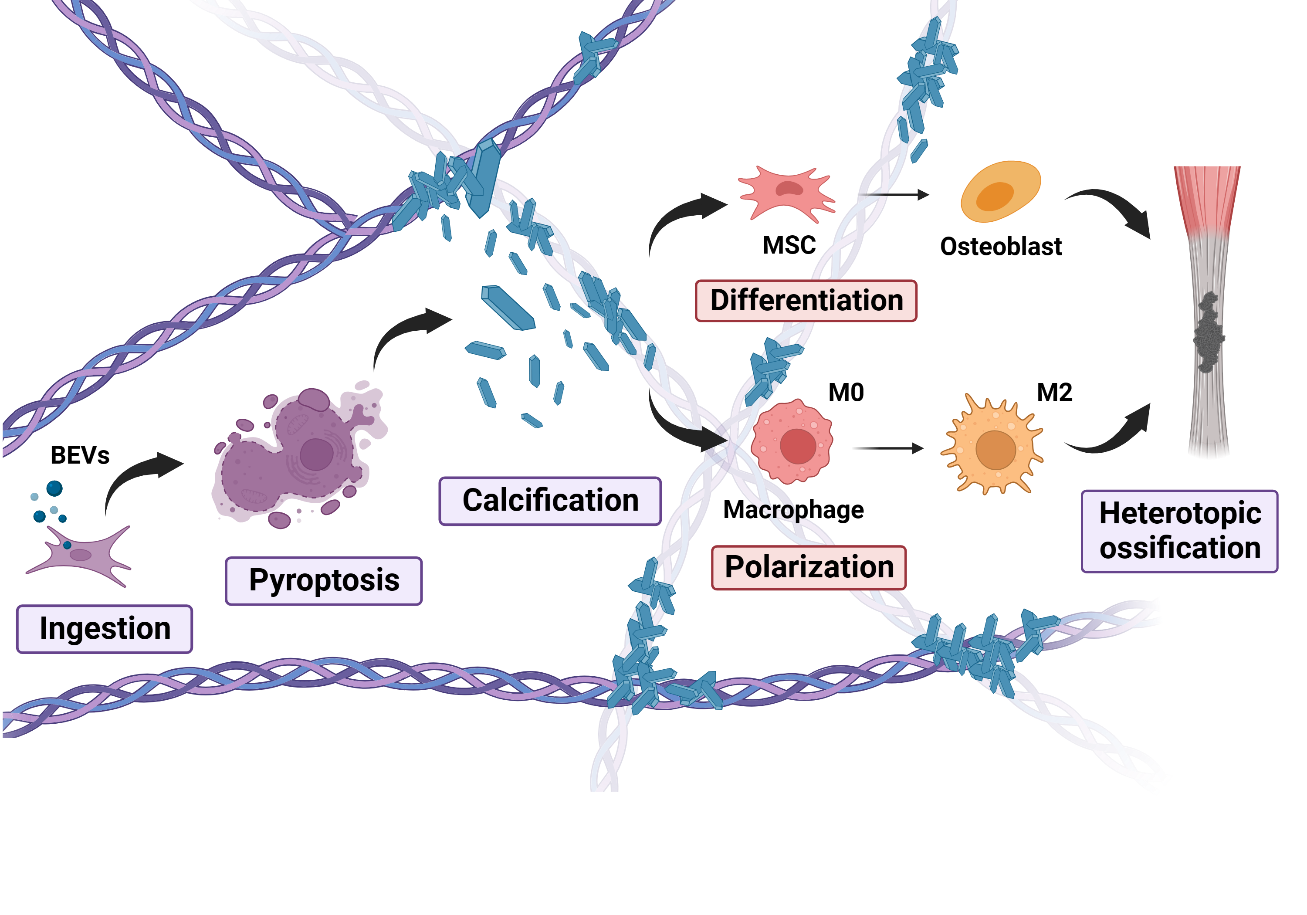
**

**Fig. S12.** Schematic of the formation of osteogenic microenvironment for NHO. BEVs induced fibroblast pyroptosis and the formation of calcification. The stiffness of the calcification triggered mesenchaymal stem cells (MSCs) osteogenic differentiation and favored the M2 polarization of macrophages, in turn leading to the ectopic bone formation.

**
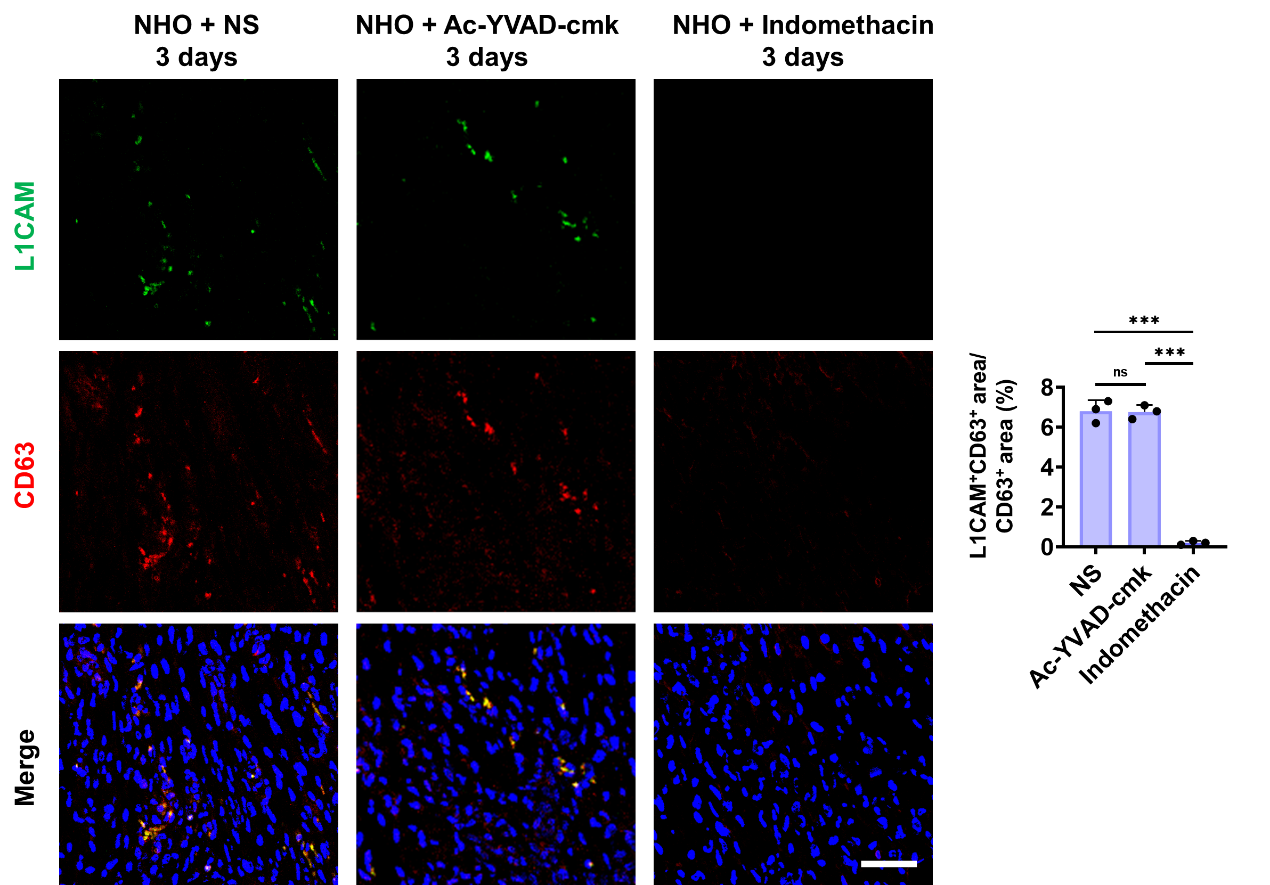
**

**Fig. S13.** Immunofluorescence microscopy of the localization of L1CAM (green) and CD63 (red) in the Achilles tendon of rats from the different groups after 3 days. Scale bar, 50 μm. Data were presented as means ± standard deviations (n = 3). Statistical analyses were performed by one-way ANOVA with post-hoc Tukey’s test. ns, no significance. ***P < 0.001.

NHO rats EV. EXCEL

**Table Supplementary 1**

**The protein carried by EV isolated from NHO rats' plasma and contrasted with EV isolated from HO rats' plasma by the mass spectrometer.**
